# Accumulation of Damaging Lipids in the Arf1‐Ablated Neurons Promotes Neurodegeneration through Releasing mtDNA and Activating Inflammatory Pathways in Microglia

**DOI:** 10.1002/advs.202414260

**Published:** 2025-02-28

**Authors:** Xu Li, Shuhan Jin, Danke Wang, Ying Wu, Xiaoyu Tang, Yufan Liu, Tiange Yao, Shoufa Han, Lin Sun, Yuetong Wang, Steven X. Hou

**Affiliations:** ^1^ Department of Cell and Developmental Biology at School of Life Sciences State Key Laboratory of Genetic Engineering Institute of Metabolism and Integrative Biology Children's Hospital Zhongshan Hospital Fudan University Shanghai 200438 China; ^2^ State Key Laboratory for Physical Chemistry of Solid Surfaces Department of Chemical Biology College of Chemistry and Chemical Engineering The Key Laboratory for Chemical Biology of Fujian Province The MOE Key Laboratory of Spectrochemical Analysis & Instrumentation Innovation Center for Cell Signalling Network Xiamen University Xiamen 361005 China

**Keywords:** Arf1 ablation, accumulation of damaging lipids, mtDNA release, neurodegeneration

## Abstract

Lipid metabolism disorders in both neurons and glial cells have been found in neurodegenerative (ND) patients and animal models. However, the pathological connection between lipid droplets and NDs remains poorly understood. The recent work has highlighted the utility of a neuron‐specific Arf1‐knockout mouse model and corresponding cells for elucidating the nexus between lipid metabolism disorders and amyotrophic lateral sclerosis (ALS) and multiple sclerosis (MS). In this study, it is found that Arf1 deficiency first induced surplus fatty acid synthesis through the AKT‐mTORC1‐SREBP1‐FASN axis, which further triggered endoplasmic reticulum (ER)‐mitochondrial stress cascade via calcium flux. The organelle stress cascade further caused mitochondrial DNA (mtDNA) to be released into cytoplasm. Concurrently, the FASN‐driven fatty acid synthesis in the Arf1‐deficient neurons might also induce accumulation of sphingolipids in lysosomes that caused dysfunction of autophagy and lysosomes, which further promoted lysosomal stress and mitochondria‐derived extracellular vesicles (MDEVs) release. The released MDEVs carried mtDNA into microglia to activate the inflammatory pathways and neurodegeneration. The studies on neuronal lipid droplets (LDs) and recent studies of microglial LDs suggest a unified pathological function of LDs in NDs: activating the inflammatory pathways in microglia. This finding potentially provides new therapeutic strategies for NDs.

## Graphical abstract

Arf1 defects activate the AKT‐mTORC1‐SREBP1‐FASN pathway to increase lipid synthesis and disrupt ER‐mitochondrial‐lysosomal homeostasis in neurons. Impaired mitochondria secrete damaged mtDNA via mitochondria‐derived extracellular vesicles, which is phagocytosed by the microglia and activated inflammation signaling. Knocking down Fasn in neurons or enhancing lysosomal function with Myriocin can rescue the neurodegenerative phenotypes, offering new therapeutic targets for neurodegenerative diseases.

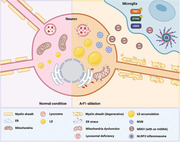

## 1 Introduction

Neurodegenerative diseases (ND) are currently one of the leading causes of human disability and death.^[^ ^1^, ^2^, ^3^ ^]^ Characteristically, NDs are often characterized by the aggregation of misfolded proteins into insoluble deposits within the central nervous system (CNS).^[^ ^4^ ^]^ The potential link between lipid droplets and neurodegeneration was first posited over a century ago, with Alois Alzheimer noting in 1907 that glial cells from the brains of Alzheimer's disease (AD) patients exhibited a pronounced accumulation of lipid droplets post‐mortem, contrasting with those from non‐diseased brains.^[^ ^5^ ^]^ Subsequent research has implicated disorders in lipid metabolism, the formation of lipid peroxides, and associated cellular damage in the pathogenesis of various NDs, including Alzheimer's disease (AD), Parkinson's disease (PD), ALS, and MS, among others.^[^ ^6^ ^]^ In addition, genetic meta‐analyses of recently diagnosed Alzheimer's patients identified several genes for lipid metabolism as novel genetic risk loci, including APOE.^[^ ^7^ ^]^ Disruptions in lipid metabolism and alterations in APOE have been observed in both neurons and glial cells across ND patients and animal models.^[^ ^5^, ^6^, ^7^, ^8^, ^9^, ^10^, ^11^, ^12^, ^13^, ^14^, ^15^, ^16^, ^17^ ^]^ Despite these findings, the causal relationship between lipid droplets and neurodegeneration has remained elusive due to the absence of an optimal experimental research framework, and the mechanistic understanding of this association remains limited until recent advancements in the field.

Recent research emanating from Wyss‐Coray's laboratory has delineated the predominantly detrimental or neurotoxic functions of microglial lipid droplets (LDs) in neurodegenerative contexts.^[^ ^9^, ^10^ ^]^ It was first found that aged mice accumulated more LD‐loaded microglia termed LD‐accumulating microglia (LDAM), the mouse LDAM are defective in phagocytosis and secrete proinflammatory cytokines.^[^ ^9^ ^]^ A recent study found that the human brains upregulated expression of LD‐related enzyme ACSL1 and accumulated LDAM, particularly in microglia of Alzheimer's disease patients with Apolipoprotein E4 (APOE4), the strongest single genome‐wide associated risk variant in AD.^[^ ^10^ ^]^ In cultured human induced pluripotent stem (iPS) cell‐derived microglia (iMG), fibrillary Aβ treatment induces ACSL1 expression and LDAM accumulation in an APOE‐dependent manner. The LDAM can further release factor induces Tau phosphorylation and neurotoxicity in neurons in an APOE‐dependent manner.^[^ ^10^ ^]^

In *Drosophila* neurons, it was found that elevated levels of reactive oxygen species (ROS) (due to aging, genetic risk, or other factors) increased lipid synthesis and elevated lipid peroxidation, which are often transferred into glial cells through an ApoE‐mediated transport mechanism to form lipid droplets (LDs) that protect neurons from lipid toxicity. However, when mitochondrial damage induces excessive elevation of ROS and defects in neuron‐glial lipid transfer, it can lead to the accumulation of oxidized lipids in neurons and extracellular spaces, thereby inducing neurodegeneration.^[^ ^12^, ^13^, ^14^, ^15^ ^]^

Our recent study found that Arf1‐mediated lipid metabolism maintains neuronal function, and its specific knockout in mouse neurons triggers a complete circuit ranging from lipid droplet accumulation in neurons to activation of glial cell neuroinflammation and peripheral immune systems to final demyelination and neurodegeneration.^[^ ^16^, ^17^ ^]^ Despite these findings, the precise molecular underpinnings by which Arf1 deficiency induces lipid metabolic perturbations and stimulates inflammatory responses in microglia, consequently precipitating neurodegeneration, remain to be elucidated.

In this study, we found that Arf1‐deficiency upregulated lipogenesis enzyme FASN to induce surplus fatty acid synthesis, which further triggered endoplasmic reticulum (ER)‐mitochondrial stress cascade via calcium flux to release mtDNA. Meanwhile, the FASN‐driven fatty acid synthesis in Arf1‐deficient neurons might also induce accumulation of sphingolipids in lysosomes to trigger MDEVs release, the released EVs carried mtDNA into microglia to activate the inflammatory pathways that promoted neurodegeneration. Inhibition of FASN or reducing accumulation of sphingolipids in lysosomes with Myriocin treatment in vivo significantly rescued the neurodegenerative phenotypes of the Arf1‐deficient mice. We also found functional activation mutation of FASN in human neurodegeneration patients, suggesting a causal role of surplus fatty acid synthesis in ND disease onset.

## 2 Results

### 2.1 Arf1 Deficiency Enhanced Lipid *de Novo* Synthesis by Activating the AKT‐mTORC1‐SREBP1‐FASN Axis

We have previously reported thatLDs were dramatically increased in the brains of the Arf1‐ablated mice compared to that of control mice.^[^ ^16^ ^]^ LDs are specialized cytosolic organelles for fatty acid storage.^[^ ^18^ ^]^ To elucidate the mechanisms by which Arf1‐deficiency induces lipid accumulation in neurons, we systematically investigated the impact of lipid uptake, secretion, and de novo lipid synthesis on this accumulation. We initiated our inquiry by employing BODIPY‐C1, C12 to assess the influence of Arf1‐deficiency on lipid uptake and secretion (Figure S1A, Supporting Information). After pre‐culturing N2a cells with BODIPY‐C1, C12 for 30 min, we found that Arf1‐deficiency did not affect lipid uptake in N2a cells. The expression levels of fatty acid transporter family proteins involved in lipid uptake were not significantly changed in the RNA‐seq of Arf1‐deficient N2a cells, which might further explain why Arf1‐deficiency did not affect lipid uptake (Figure S1B‐C, Supporting Information). Furthermore, there was no significant alteration in the fluorescence intensity of BODIPY‐C1, C12 in N2a cells after an 8h incubation period, and the change in fluorescence intensity was comparable to the initial levels, suggesting that Arf1‐deficiency did not significantly influence lipid secretion (Figure S1D‐E, Supporting Information).

**Figure 1:**
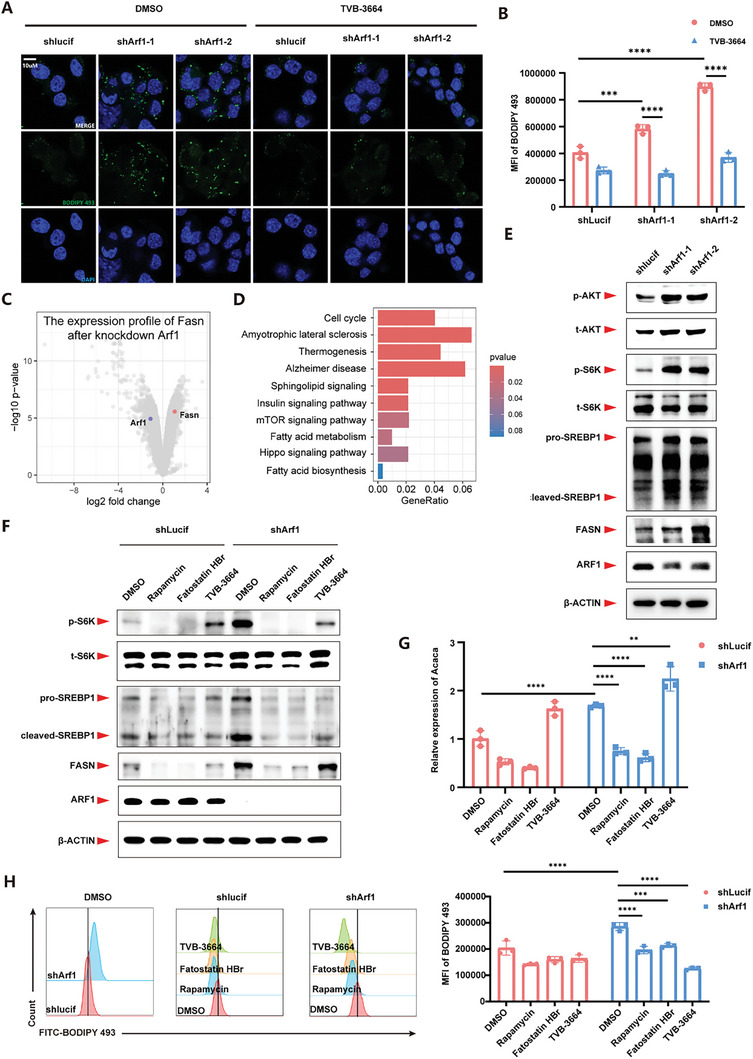
Arf1 deficiency enhanced lipid de novo synthesis by activating the mTORC1‐SREBP1‐FASN axis to trigger lipid accumulation. A) Representative BODIPY 493 images of control or Arf1‐deficient N2a cells treated with DMSO or TVB‐3664. B) Flow cytometry of BODIPY 493 from control or Arf1‐deficient N2a cells treated with DMSO or TVB‐3664. The mean fluorescence intensity of BODIPY 493 is shown. n = 3 per group. C) Volcano plot analysis of RNA‐seq data obtained from Arf1‐deficient N2a cells vs. vehicle control, n = 3 per group. D) KEGG enrichment analysis of RNA‐seq data obtained from Arf1‐deficient N2a cells vs. vehicle control, n = 3 per group. E) Western blots of N2a cells as indicated. F) Western blots of N2a cells to verify the regulation of mTORC1‐SREBP1 pathway to FASN. G) Relative expression of Acaca, another SREBP1 downstream gene. n = 3 per group. H) Flow cytometry of BODIPY 493 from control or Arf1‐deficient N2a cells treated with DMSO, Rapamycin, Fatostatin HBr, or TVB‐3664. The representative FACS plots and mean fluorescence intensity of BODIPY 493 are shown. n = 3 per group. Data are shown as the mean ± SEM. *P < 0.05, **P < 0.01. ***P < 0.001. ****P < 0.0001 by two‐way ANOVA with Holm Šídák's multiple comparisons test. See also Figure S1 (Supporting Information).

Subsequently, we used TVB‐3664, an inhibitor of FASN (a key enzyme in *de novo* lipid synthesis), to block *de novo* lipid synthesis and found that it could significantly rescue the lipid accumulation caused by Arf1‐deficiency and showed a dose dependence on TVB‐3664 (**Figures** **1**A‐B; S1F, Supporting Information). Further, we found that Arf1‐deficiency could increase the expression of FASN (Figures 1C, S1H, and Figure S10D, Supporting Information). Arf1 was reported to function as a negative regulator of mTORC2, and Arf1 defects could significantly activate AKT.^[^ ^19^, ^20^ ^]^ We speculated that Arf1‐deficiency might upregulate the expression of FASN by activating the AKT‐mTORC1‐SREBP1 signaling pathway. Therefore, we examined and found that phosphorylated AKT, phosphorylated S6K (used to characterize mTORC1 activity), and cleaved mature SREBP1 were significantly increased in the Arf1 knockdown cells compared to those in the control cells. The pathways and genes related to the AKT‐mTORC1‐SREBP1 axis were enriched in the Arf1‐deficient cells, suggesting that Arf1‐deficiency significantly activated the AKT‐mTORC1‐SREBP1 signaling axis (Figures 1D‐E, S1G‐I, Supporting Information). To verify the regulatory impact of this signaling pathway on FASN expression, we utilized Rapamycin to inhibit mTORC1, Fotastain HBr to inhibit SREBP1, and TVB‐3664 to inhibit FASN, respectively. As a result, we observed that inhibition of the AKT‐mTORC1‐SREBP1 signaling pathway completely blocked the upregulation of FASN and ACACA induced by Arf1‐deficiency (Figure 1F‐G). Moreover, the lipid accumulation was significantly reduced by inhibition of the AKT‐mTORC1‐SREBP1 signaling pathway (Figure 1H). Collectively, our data suggest that Arf1‐deficiency induced lipid accumulation, potentially through the activation of the AKT‐mTORC1‐SREBP1 signaling pathway, leading to increased FASN expression and subsequent *de novo* lipid synthesis.

### 2.2 Elevated Lipid Levels Triggered the Stress Cascade of ER‐Mitochondria Organelles

**Figure 2:**
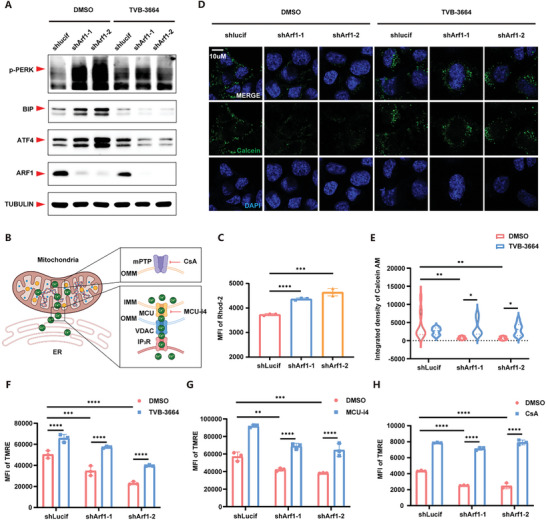
Elevated lipid levels triggered the stress cascade of ER‐mitochondria organelles. A) ER stress associated markers’ expression of N2a cells by western blots. B) Schematic of ER‐mitochondria Ca^2+^ transportation. C) Flow cytometry of Rhod‐2 from control or Arf1‐deficient N2a cells. The mean fluorescence intensity of Rhod‐2 is shown. n = 3 per group. D) Representative Calcein AM images of N2a cells. E) Quantification of the integrated fluorescence intensity of Calcein AM (D). n = 13, 10, 14, 8, 13, 12 respectively. F) Flow cytometry of TMRE from control or Arf1‐deficient N2a cells treated with DMSO or TVB‐3664. The mean fluorescence intensity of TMRE is shown. n = 3 per group. G) Flow cytometry of TMRE from control or Arf1‐deficient N2a cells treated with DMSO or MCU‐i4. The mean fluorescence intensity of TMRE is shown. n = 3 per group. H) Flow cytometry of TMRE from control or Arf1‐deficient N2a cells treated with DMSO or CsA. The mean fluorescence intensity of TMRE is shown. n = 3 per group. Data are shown as the mean ± SEM. *P < 0.05, **P < 0.01. ***P < 0.001. ****P < 0.0001 by two‐way ANOVA with Holm Šídák's multiple comparisons test. See also Figure S2 (Supporting Information).

Next, we investigated the mechanism by which accumulated lipids exerts a deleterious effect on neuronal function. Accumulated saturated fatty acid is reported to induce ER stress through lipotoxity.^[^ ^21^, ^22^ ^]^ Upregulation or gain‐of‐function mutation of FASN, which could increase fatty acid synthesis and accumulation, could also induce ER stress.^[^ ^23^, ^24^ ^]^ Thus, we hypothesized that elevated fatty acid synthesis induced by Arf1‐deficiency might cause ER stress. As shown in (**Figure** **2**A), severe ER stress was induced by Arf1‐deficiency, which could be eliminated by blocking lipid synthesis.

As a pivotal organelle for calcium sequestration, the endoplasmic reticulum (ER) plays a critical role in modulating mitochondrial calcium homeostasis, thereby influencing the transition of the mitochondrial permeability transition pore (mPTP). This regulatory process is mediated through the IP3R‐VDAC1‐MCU calcium channel complex (Figure 2B). We found that Arf1‐deficiency led to a significant accumulation of mitochondrial calcium, accompanied by mPTP opening and loss of mitochondrial membrane potential (MMP) (Figure 2C‐F, and Figure S10A, Supporting Information). The inhibition of FASN could inhibit ER stress and block mPTP opening (Figure 2A,D,E). We speculated that an imbalance in mitochondrial calcium homeostasis might trigger mPTP opening and lead to the loss of mitochondrial membrane potential. Indeed, we found that after inhibiting FASN via TVB‐3664, blocking ER‐mitochondria calcium transportation via MCU‐i4, and closing mPTP via CsA, the loss of MMP induced by Arf1‐deficiency could be dramatically reversed (Figure 2F‐H, and Figure S2A‐C, Supporting Information). In summary, these findings suggest that the lipids accumulation, resulting from Arf1‐deficiency, may be associated with ER stress, which could disrupt mitochondrial calcium homeostasis and consequently contribute to mitochondrial dysfunction.

### 2.3 Dysfunctional Mitochondria Induced the Leakage of Damaged mtDNA and Promoted Microglial Inflammation

**Figure 3:**
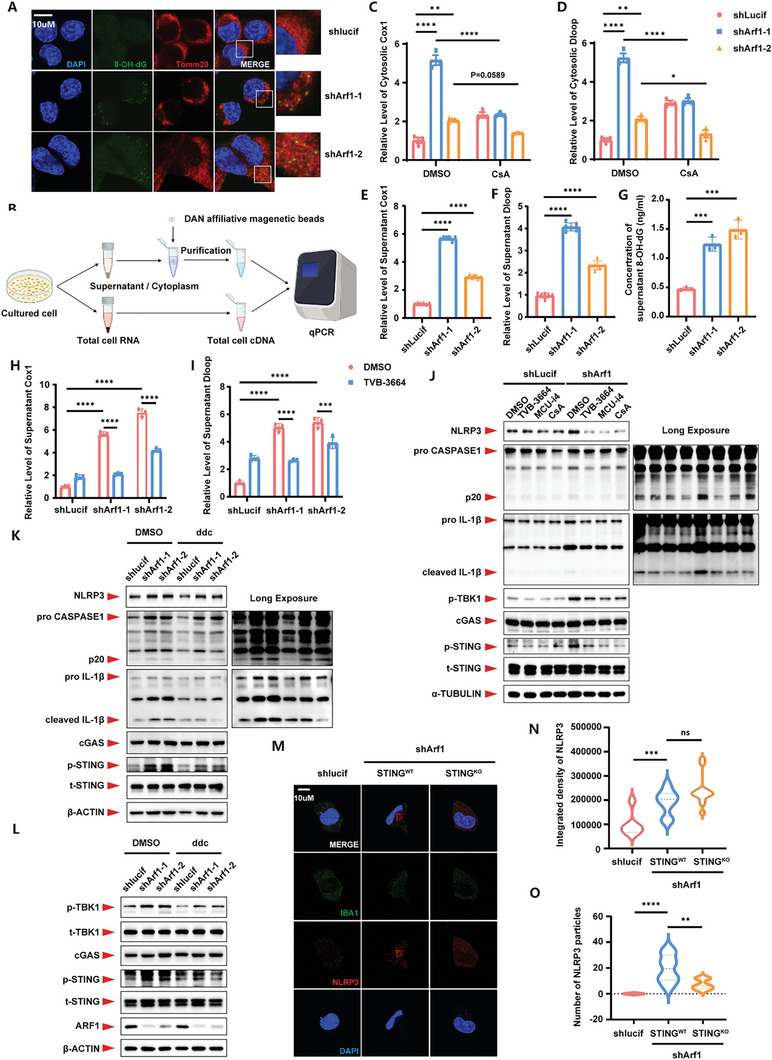
Dysfunctional mitochondria induced oxidative damaged mtDNA release and induced microglia inflammation. A) Representative 8‐OH‐dG (Green) and Tomm20 (Red) images of control or Arf1‐deficient N2a cells. B) Schematic of cytosolic and supernatant DNA quantification from control or Arf1‐deficient N2a cells. C) Relative abundance of cytosolic Cox1 from control or Arf1‐deficient N2a cells treated with DMSO or CsA. n = 3 per group. D) Relative abundance of cytosolic Dloop from control or Arf1‐deficient N2a cells treated with DMSO or CsA. n = 3 per group. E) Relative abundance of supernatant Cox1 from control or Arf1‐deficient N2a cells. n = 3 per group. F) Relative abundance of supernatant Dloop from control or Arf1‐deficient N2a cells. n = 3 per group. G) Secreted 8‐OH‐dG was measured from control or Arf1‐deficient N2a cells derived supernatant by ELISA. n = 3 per group. H) Relative abundance of supernatant Cox1 from control or Arf1‐deficient N2a cells treated with DMSO or TVB‐3664. n = 3 per group. I) Relative abundance of supernatant Dloop from control or Arf1‐deficient N2a cells treated with DMSO or TVB‐3664. n = 3 per group. J) Western blots of Eoc20 cells co‐cultured with conditional medium from control or Arf1‐deficient N2a cells treated with DMSO, TVB‐3664, MCU‐i4, or CsA. K) Western blots of Eoc20 cells co‐cultured with conditional medium from control or Arf1‐deficient N2a cells treated with DDC. L) Western blots of control or Arf1‐deficient N2a cells treated with DDC. M) Representative IBA1 (Green) and NLRP3 (Red) images of STING^WT^ or STING^KO^ primary microglia co‐cultured with conditional medium from control or Arf1‐deficient N2a cells. N) Quantification of the integrated fluorescence intensity of NLRP3 (M). n = 5, 8, 12 respectively. O) Quantification of the number of NLRP3 particles (M). n = 5, 8, 12 respectively. Data are shown as the mean ± SEM. *P < 0.05, **P < 0.01. ***P < 0.001. ****P < 0.0001 by two‐way ANOVA with Holm Šídák's multiple comparisons test. See also Figure S3 (Supporting Information).

Mitochondrial dysfunction has been previously implicated in the excessive generation of reactive oxygen species (ROS) and the oxidative damage of mtDNA, which subsequently translocates through the mPTP to instigate inflammatory responses.^[^ ^25^, ^26^ ^]^ However, how neuronal mtDNA triggers neuroinflammation in glial cells remains unknown. We found that Arf1‐deficiency induced massive oxidative DNA damage, marked by 8‐OH‐dG, in mitochondria (**Figure** **3**A). By extracting free DNA from the cytoplasm, we found that Arf1‐deficiency upregulated the level of COX1 and Dloop (mitochondria‐specific genes) (Figure 3B‐D; Figures S3A and S10B, Supporting Information), and robustly activated the cGAS‐STING signaling pathway in N2a cells (Figures S3C and S10E, Supporting Information). Further, we detected that the oxidatively damaged mtDNA (8‐OH‐dG) was secreted outside neurons and released into the supernatant (Figure 3B,E‐G; Figure S3B, Supporting Information). Moreover, blocking mPTP opening resulted in a significant reduction of free mtDNA in the cytoplasm (Figure 3B‐C). The mtDNA in the supernatant was also significantly reduced after inhibition of FASN (Figure 3H‐I).

Our previous study found that Arf1‐deficiency induced intense neuroinflammation in microglia.^[^ ^16^ ^]^ Thus, we hypothesized that the secreted mtDNA might play an essential role in the inflammatory activation of microglia. By culturing N2a‐derived conditional medium (CM) with Eoc20, we found that CM from the Arf1‐deficient N2a cells significantly activated the cGAS‐STING signaling pathway and NLRP3 inflammasome in Eoc20 (Figure 3J). The transfection of normal mtDNA into Eco20 could also trigger the inflammatory response to some extent (Figure S3D, Supporting Information). Improving mitochondrial function by inhibiting FASN, blocking ER‐mitochondrial calcium transportation, and inhibiting the opening of mPTP, could significantly block the activation of the inflammatory pathways in Eoc20 (Figure 3J). To further understand the role of mtDNA in activating inflammatory pathways in neurons and microglia, we eliminated mtDNA using DDC. As a result, we found that the inflammatory pathways in both N2a and Eoc20, cultured with CM, were significantly inhibited by DDC treatment (Figure 3K‐L). We previously reported that knocking out NLRP3 can also alleviate the neurodegeneration induced by Arf1‐deficiency, and STING was reported to have the ability to regulate the NLRP3 inflammasome formation. So, we cultured STING^WT^ or STING^KO^ primary microglia with CM and found that STING‐deficiency made assembled NLRP3 inflammasome decamerisation (Figure 3M‐N). These results suggest that mtDNA plays a crucial role in the inflammatory activation of microglia.

### 2.4 Mitochondria‐Derived Extracellular Vesicles Mediated the Transportation of mtDNA and Induced Microglial Inflammation

**Figure 4:**
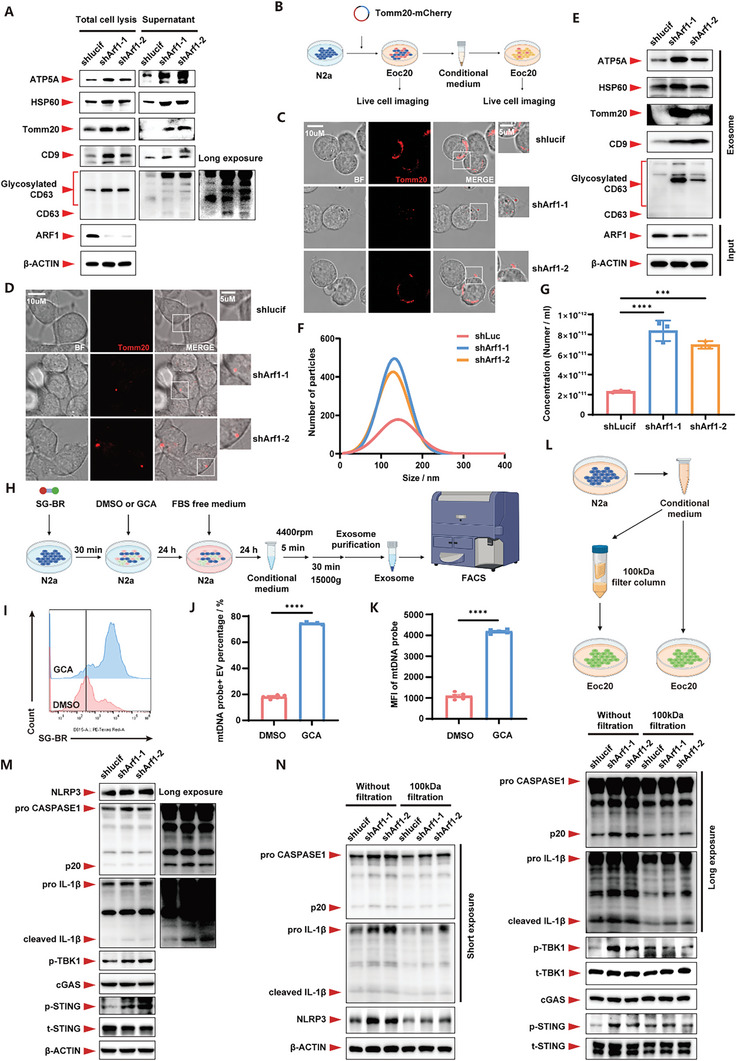
Mitochondria‐derived exosomes mediated the transportation of mtDNA and induced microglial inflammation. A) Western blots of N2a cells derived supernatant protein as indicated. B) Schematic of in vitro monitoring mitochondria secretion. C) Representative Tomm20‐mCherry images of control or Arf1‐deficient N2a cells. D) Representative Tomm20‐mCherry images of Eoc20 cells treated with the conditional medium from control or Arf1‐deficient N2a cells. E) Western blots of exosomes from control or Arf1‐deficient N2a cells. F) Particle size analysis of exosomes from control or Arf1‐deficient N2a cells. G) Concentration of exosomes from control or Arf1‐deficient N2a cells. n = 3 per group. H) Schematic of mtDNA labeled exosome extraction from DMSO or GCA treated N2a cells. I) Flow cytometry of SG‐BR of exosomes from DMSO or GCA treated N2a cells. J) The quantification of the percent of SG‐BR positive exosome. n = 3 per group. K) The quantification of the mean fluorescence intensity of SG‐BR. n = 3 per group. L) Schematic of clearing the exosomes and high molecular weight proteins form conditional medium. M) Western blots of Eoc20 cells treated with exosomes from control or Arf1‐deficient N2a cells. N) Western blots of Eoc20 cells co‐cultured with conditional medium from control or Arf1‐deficient N2a cells filtered with or without 100kDa filtration. Data are shown as the mean ± SEM. *P < 0.05, **P < 0.01. ***P < 0.001. ****P < 0.0001 by two‐way ANOVA with Holm Šídák's multiple comparisons test. See also Figure S4 (Supporting Information).

We conducted further investigations to elucidate the mechanism of mtDNA transfer from neurons to microglia. Utilizing immunofluorescence staining for the mitochondrial outer membrane marker Tomm20 and DNA, we identified vesicles positive for both Tomm20 and DNA outside Arf1‐deficient cells (Figure S4A, Supporting Information), suggesting that mtDNA possibly secreted outside of the cell through mitochondrial‐derived extracellular vesicles (MDEVs). So, we purified proteins from supernatants of cell culture and found that there were a large number of mitochondrial components, such as ATP5A and Tomm20, as well as extracellular vesicles (EV)‐associated proteins, such as CD9 and CD63 in the supernatants of the Arf1‐deficient cells (**Figure** **4**A). To visualize MDEV secretion, we performed live‐cell imaging after transfection of the Tomm20‐mCherry plasmid into N2a cells. We found that Tomm20‐containing vesicles appeared in the periphery and outside of the Arf1‐deficient cells (Figure 4B‐C, Figure S4B,D, Supporting Information). Subsequently, we cultured Eoc20 with the conditional medium of N2a transfected with the Tomm20‐mCherry plasmid and found mCherry signals in Eoc20, suggesting that Eoc20 phagocytosed the Tomm20‐containing vesicles (Figure 4D, Figure S4C, Supporting Information). We purified EVs from N2a‐derived supernatants to more precisely depict the composition of these Tomm20 containing vesicles. We found that Arf1‐deficiency significantly increased the release of EVs. The purified EVs fractions had EV marker proteins, such as CD9 and CD63, as well as up‐regulated mitochondrial fractions, suggesting that Arf1 deficiency facilitated the release of MDEVs (Figure 4E‐G; Figure S4E, Supporting Information).

To explore whether the MDEVs contain the mtDNA that we previously detected in cell culture supernatants, we pre‐labeled mtDNA in N2a using a mtDNA‐specific probe.^[^ ^27^ ^]^ After treating cells with the Arf1 inhibitor GCA, we collected EVs from supernatants by flow cytometry and found a significant increase in mtDNA signals in the EVs released from the Arf1‐deficient cells (Figure 4I‐K). To further investigate whether the MDEVs‐carried mtDNA can activate inflammation or not, we treated Eoc20 with the purified EVs and found that the relevant inflammatory pathways were dramatically activated (Figure 4M). Moreover, after removing EVs and proteins from the conditional medium using a protein concentration column of 100 kDa, we found that the inflammatory activation in microglia associated with Arf1 deficiency was remarkably blocked (Figure 4L,N). Our findings implied that mtDNA released from neurons via MDEVs was phagocytosed into microglia and activated the inflammatory pathways.

### 2.5 Accumulation of Sphingolipids in Lysosomes Caused Lysosomal Dysfunction and Induced Multivesicular Body Formation, and Facilitated MDEV Secretion

**Figure 5:**
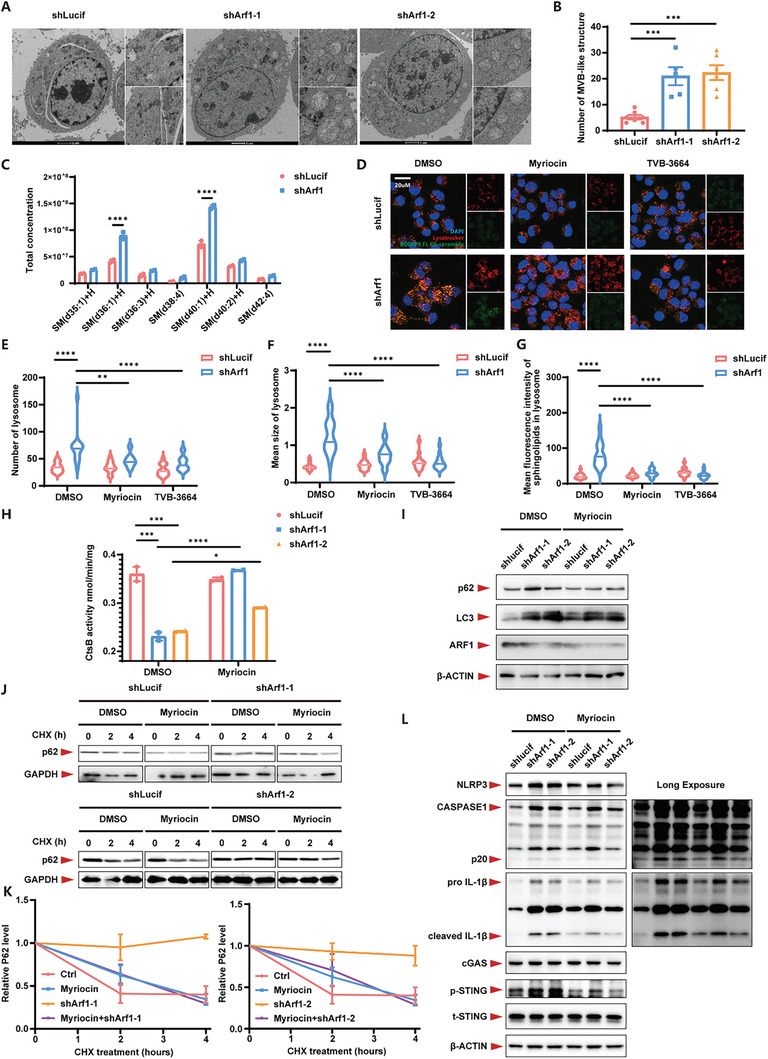
Accumulation of sphingolipids in lysosomes causes lysosomal dysfunction and induces multivesicular body formation, facilitating exosome secretion. A) Representative transmission electron microscopy images of control or Arf1‐deficient N2a cells. B) Quantification of the number of MVB‐like structures (A). n = 7, 5, 6 respectively. C) The level of multi‐type sphingolipids in lysosomes from control or Arf1‐deficient N2a cells. n = 2 per group. D) Representative BODIPY FL C_5_‐ceramide (green) and lysosome (red) images of control or Arf1‐deficient N2a cells. E) Quantification of the number of lysosomes (D). n = 15 per group. F) Quantification of the mean size of lysosomes (D). n = 15 per group. G) Quantification of lysosomal accumulated sphingolipids (D). n = 15 per group. H) The activity of lysosomal CtsB. n = 2 per group. I) Western blots of autophagy flux from control or Arf1‐deficient N2a cells treated with DMSO or Myriocin. J) Western blots of protein degradation rate from control or Arf1‐deficient N2a cells treated with DMSO or CHX. K) Quantification of protein degradation rate (J). n = 3 per group. L) Western blots of Eoc20 cells co‐cultured with conditional medium from control or Arf1‐deficient N2a cells treated with DMSO or Myriocin. Data are shown as the mean ± SEM. *P < 0.05, **P < 0.01. ***P < 0.001. ****P < 0.0001 by two‐way ANOVA with Holm Šídák's multiple comparisons test. See also Figure S5 (Supporting Information).

In pathological situations, the release of EVs is associated with the autophagy‐degradation pathway. Abnormal mitochondria form autophagic vesicles mediated by Parkin‐PINK1, followed by the further generation of MVBs, also known as late endosomes. With normal lysosomal function, MVBs fuse with lysosomes to carry out degradation, but in the case of abnormal lysosomal function, MVBs release their components as EVs.^[^ ^28^ ^]^ By electron microscopy analysis, we observed the emergence of extensive MVBs in the Arf1‐deficient cells (**Figure** **5**A‐B). We speculated that the disordered lipid metabolism in the Arf1‐deficient cells might also affect lysosomal activity, which may contribute to MVB formation and EV release. Through analyzing the lipidomic of multiple organelles, we found that Arf1‐deficiency specifically induced accumulation of sphingolipids in lysosomes (a phenomenon that has been linked to autophagy and lysosomal dysfunction^[^ ^29^ ^]^), and the finding was confirmed by imaging of both sphingolipids and lysosomes (Figure 5C‐D,G; Figure S5A‐C, Supporting Information). The accumulation of sphingolipids in lysosomes was accompanied by a marked increase in the number and size of lysosomes (Figure 5E‐F). To further clarify the function of lysosomes in the Arf1‐deficient cells, we tested the activity of CtsB (a lysosomal acid hydrolase). We found that Arf1‐deficiency significantly inhibited CtsB activity, suggesting impaired lysosomal function (Figure 5H; Figure S10C, Supporting Information). Myriocin, an inhibitor of serine palmitoyl‐transferase (SPT, the key enzyme of sphingolipids *de novo* synthesis), was reported to have the ability to boost autophagy.^[^ ^30^ ^]^ To examine the role of sphingolipids, we blocked sphingolipid synthesis using Myriocin or *de novo* lipid synthesis using TVB‐3664, both of which significantly rescued sphingolipid accumulation in lysosomes and reduced lysosome quantity and size (Figure 5D‐G), suggesting that FASN‐driven fatty acid synthesis might also induce accumulation of sphingolipids in lysosomes of the Arf1‐deficient neurons. Moreover, CtsB activity was significantly restored after Myriosin treatment (Figure 5H).

Lysosomal dysfunction affects autophagy and protein degradation. We observed that Arf1 defects were correlated with impaired autophagic flux and protein homeostasis, as indicated by an increase in p62 and LC3 levels and a reduced rate of protein degradation, which was rescued by inhibiting sphingolipid synthesis with Myriocin (Figure 5I‐K). Subsequently, we cultured Eoc20 with the conditional medium of the Myriocin‐treated N2a and found that activation of the inflammatory pathways in Eoc20 could be significantly inhibited after improving lysosomal function to enhance autophagy with Myriocin treatment (Figure 5L). These data suggest that enhancing autophagy with Myriocin treatment may be a therapeutic option to treat relative NDs.

Our results together suggest that Arf1‐deficiency in neurons induced two biological processes. One the one hand, Arf1 ablation elevated fatty acid synthesis via the AKT‐mTORC1‐SREBP‐FASN axis that further triggered organelle stress cascade to release ROS, mtDNA into the cytoplasm; on the other hand, FASN‐driven fatty acid synthesis in the Arf1‐deficient neurons might also induce accumulation of sphingolipids in lysosomes that caused dysfunction of autophagy and lysosomes, which further promoted lysosomal stress and MDEV release, the released MDEVs carried mtDNA into microglia to activate the inflammatory pathways.

### 2.6 Blockade of Fatty Acid Synthesis Reversed Neuronal Degeneration

**Figure 6:**
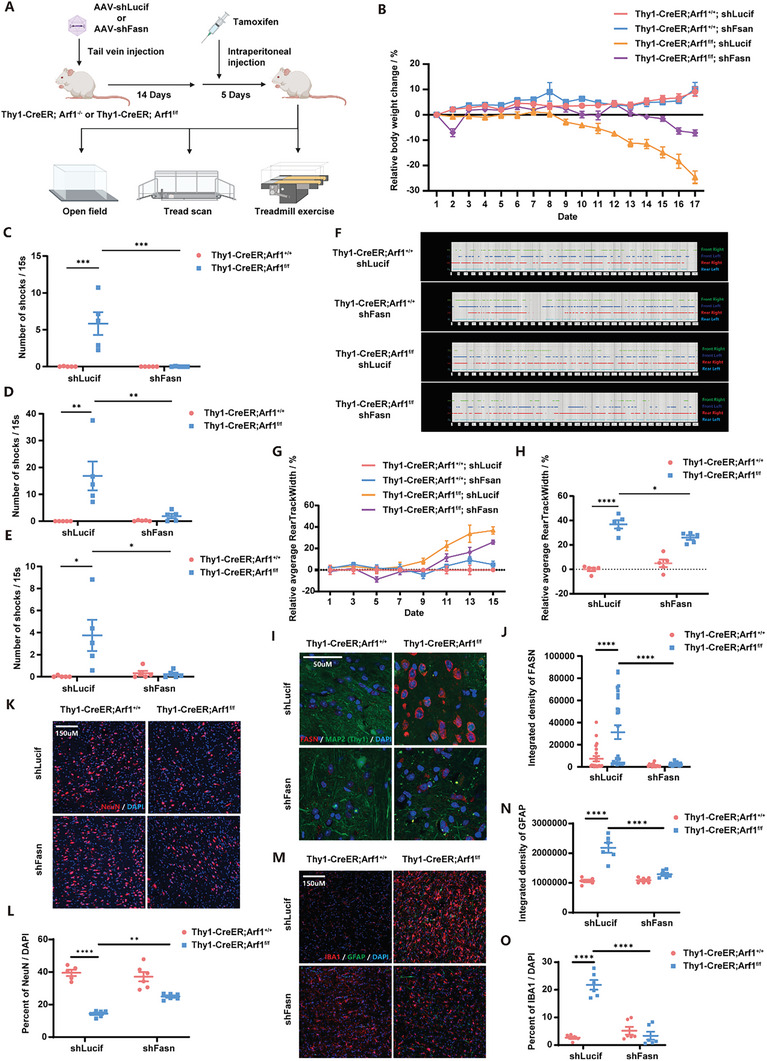
Blockade of fatty acid synthesis reverses neuronal degeneration A) Schematic of AAV injection and behavioral experiments of mice. B) Relative body weight change of mice compared to the first day. n = 5 per group. C) Number of shocks per 15 s at 5 m min^−1^ speed on the 17th day. n = 5 per group. D) Number of shocks per 15 s at 10 m min^−1^ speed on the 17th day. n = 5 per group. E) Number of shocks per 15 s at 15 m min^−1^ speed on the 13th day. n = 5 per group. F) Representative images of mice footprint map. G) Relative average rear‐track width of mice compared to the control group. n = 5 per group. H) Relative average rear‐track width of mice compared to the control group on the 15th day. n = 5 per group. I) Representative MAP2/Thy1 (green) and FASN (red) images of control or Arf1‐deficient mouse injected with shLucif or shFasn AAV. J) Quantification of the integrated intensity of FASN in MAP2/Thy1 positive cells (I). n = 22, 26, 15, 25 respectively. K) Representative NeuN (red) images of control or Arf1‐deficient mouse injected with shLucif or shFasn AAV. L) Quantification of the percent of neurons (K). n = 5, 6, 6, 6 respectively. M) Representative IBA1 (red) and GFAP (green) images of control or Arf1‐deficient mouse injected with shLucif or shFasn AAV. N) Quantification of the integrated intensity of GFAP (M). n = 6 per group. O) Quantification of the activated microglia marked by IBA1(M). n = 6 per group. Data are shown as the mean ± SEM. *P < 0.05, **P < 0.01. ***P < 0.001. ****P < 0.0001 by two‐way ANOVA with Holm Šídák's multiple comparisons test. See also Figures S6 and S7 (Supporting Information).

Likewise, we further validated our in vitro findings in mice. We first injected AAV‐shLuciferase or AAV‐shFasn into Thy1‐CreERT2; Arf1^+/+^ or Thy1‐CreERT2; Arf1^f/f^ mice via tail vein and waited for 14 days for expression of the shRNA, followed by intraperitoneal injections of tamoxifen for 5 consecutive days to induce Arf1 knockout. Then, we recorded changes of the mouse body weights and performed three behavioral experiments, including open field, tread scan, and treadmill exercise (**Figure** **6**A). We found that knockdown of *Fasn* significantly rescued the loss of body weight induced by Arf1‐deficiency (Figure 6B; Figure S6A‐B, Supporting Information) and was also very effective in improving the locomotor performance of mice, as evidenced by a significant decrease in the number of electroshocks in Thy1‐CreERT2; Arf1^f/f^; shFasn mice compared to Thy1‐CreERT2; Arf1^f/f^; shLuciferase mice at 5 m min^−1^, 10 m min^−1^, and 15 m min^−1^, respectively (Figure 6C‐E; Figure S6C‐K, Supporting Information). Through a tread scan, we also found that the movement posture of Thy1‐CreERT2; Arf1^f/f^; shFasn mice was significantly improved (Figure 6F, Video S1, Supporting Information). The relative rear‐track‐width of Thy1‐CreERT2; Arf1^f/f^; shFasn mice were decreased, which indicates improved gait in mice (Figure 6G‐H). In the open‐field experiment, the distance of mouse movement showed a slight improvement, but there was no significant difference (Figure S7A‐E, Supporting Information). We considered that this could be due to motor impairment of the Thy1‐CreERT2; Arf1^f/f^; shFasn mice, causing them to be more inclined to rest without external pressure. After completing the behavioral experiments, we also stained the brain tissues of the mice, firstly for FASN, and verified the knockdown efficiency of FASN (Figure 6I,J). The most critical inflammatory indicators of neurodegeneration induced by Arf1 defects are the loss of neurons and the activation of microglia and astrocytes, and we found that the Thy1‐CreERT2; Arf1^f/f^; shFasn mice showed a significant increase in the number of neurons and a dramatic decrease in the amount of activated microglia and astrocytes as compared to those of Thy1‐CreERT2; Arf1^f/f^; shLuciferase mice (Figure 6K‐O).

### 2.7 Reducing Level of Sphingolipid and Restoring Lysosomal Function with Myriocin Treatment Reversed Neuronal Degeneration

**Figure 7:**
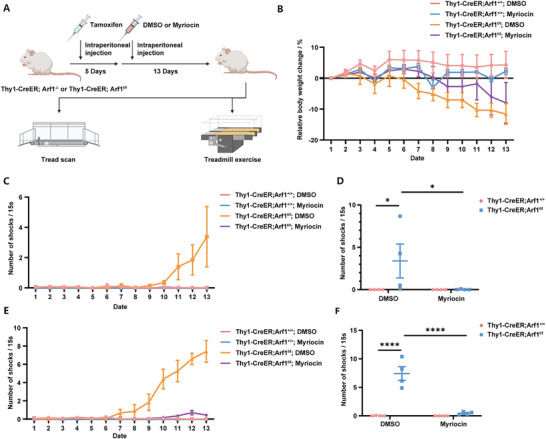
Boosting autophagy reverses neuronal degeneration. A) Schematic of DMSO or Myriocin administration and behavioral experiments of mice. B) Relative body weight change of mice compared to the first day. n = 4 per group. C) Number of shocks per 15 s at the speed of 10 m min^−1^. n = 4 per group. D) Number of shocks per 15 s at the speed of 10 m min^−1^ on the 13th day. n = 4 per group. E) Number of shocks per 15 s at the speed of 15 m min^−1^. n = 4 per group. F) Number of shocks per 15 s at the speed of 15 m min^−1^ on the 13th day. n = 4 per group. Data are shown as the mean ± SEM. *P < 0.05, **P < 0.01. ***P < 0.001. ****P < 0.0001 by two‐way ANOVA with Holm Šídák's multiple comparisons test. See also Figure S8 (Supporting Information).

After validating the role of FASN in neurodegeneration induced by Arf1‐deficiency, we also sought to explore in depth the therapeutic effects of Myriocin, which can reduce sphingolipid levels in the brain by intraperitoneal injection and has potential prospects as a drug. After knockout of Arf1 by intraperitoneal injection of tamoxifen for 5 consecutive days, we intraperitoneally injected Myriocin at a dose of 1 mg kg^−1^ into mice daily and performed two behavioral experiments, including tread scan and treadmill exercise, and recorded the changes in body weights of the mice (**Figure** **7**A). Myriocin injection showed some weight salvage effect, but there was no significant difference (Figure 7B). In the treadmill exercise, there was no significant difference between the four groups of mice at a speed of 5 m min^−1^ (Figure S8A‐D, Supporting Information). During higher intensity treadmill exercise, the number of electroshocks per 15 s in Thy1‐CreERT2; Arf1^f/f^ mice at 10 m min^−1^ and 15 m min^−1^ increased significantly over time, while it was considerably reduced after treating Thy1‐CreERT2; Arf1^f/f^ mice with Myriocin (Figure 7C‐F; Figure S8E‐H, Supporting Information). By analyzing the gait of the mice, we found that Myriocin treatment also improved the locomotion of the mice to a certain extent (Video S2, Supporting Information).

Considering that the TVB‐2664 (inhibitor of FASN) and Myriocin (autophagy activator) have been used in several clinical trials, including nonalcoholic fatty liver disease (NAFLD) and glioblastoma,^[^ ^31^, ^32^, ^33^, ^34^ ^]^ our results were inspiring. Together, our study emphasizes the importance of neuroinflammation induced by dysregulated FASN‐mediated *de novo* fatty acid synthesis in NDs and the potential therapeutic application of the indicated drugs. However, detailed pharmacodynamic analysis remains to be determined.

### 2.8 Functional Activation of FASN is Associated with Human NDs

Due to the limitation of ND patient samples, we could not analyze the expression and activity of FASN; the clinical evidence of the vital role of FASN‐mediated *de novo* fatty acid synthesis has been discovered in several large‐scale genetic variant studies in ND patients.^[^ ^35^, ^36^, ^37^, ^38^ ^]^ The genetic variants of FASN showed a causal correlation with ND incidence, especially R1819W^23^ and A1089A^37^ (Figure S9A‐B, Supporting Information).

## 3 Discussion

### 3.1 LDs and NDs

Accumulation of aberrant lipid droplets and specific dysregulated lipid species have been observed in the neurons of patients with neurodegenerative diseases,^[^ ^39^, ^40^ ^]^ microglia,^[^ ^8^, ^9^, ^10^ ^]^ and astrocytes.^[^ ^41^ ^]^ The causal association between lipid droplets and neurodegeneration has not been established due to the lack of an ideal experimental research system. Recent studies of LDs in microglia^[^ ^9^, ^10^ ^]^ and our studies of LDs in neurons^[^ ^16^, ^17^ ^]^ shed new light on the connection. The studies from Wyss‐Coray's laboratory found that microglia in aged mouse brains or in Alzheimer's disease patients accumulated LDs to form more LD‐loaded dysfunctional microglia termed LD‐accumulating microglia (LDAM); the LDAM are inflammatory and release factors to induce Tau phosphorylation and neurotoxicity in neurons.^[^ ^9^, ^10^ ^]^ Interestingly, another ND protein, alpha‐synuclein (α‐Syn), also interacts with LDs and activates an inflammatory pathway. In the *Drosophila* PD model, it was demonstrated that overexpression of α‐Syn promoted LD accumulation in neurons, and LDs could also enhance α‐Syn aggregation^[^ ^42^ ^]^ and induce microglial NLRP3 inflammasome activation.^[^ ^43^ ^]^ In addition, a previous study reported that NLRP3 activation in microglia induces neuronal tau hyperphosphorylation and aggregation in an IL‐1β‐dependent manner.^[^ ^44^ ^]^ This information together suggests such a model that accumulated LDs (either neuron or microglia) in NDs may first activate the inflammatory pathways in microglia that then release factors (such as IL‐1β and other) to induce neuronal Tau phosphorylation and other hallmarks of neurodegeneration.

Our recent publications and this study illustrated a consistent but slightly different picture with a detailed molecular mechanism. We identified a neuro‐immune circuit triggered by Arf1 deficiency with microglia activation being the key node. In brief, we discovered that the absence of Arf1 in neurons releases various neuroinflammatory triggers, such as oxidized lipids, ATP, and the recently discovered mtDNA, which can be phagocytosed by microglia and activate inflammatory responses to produce IL‐1β. The IL‐1β, along with chemokines, recruited and activated γδT cells in the meninges, and the INF‐γ released from γδT cells activated a microglia‐A1 astrocyte‐C3‐neuron C3aR circuit. In Wang's study, it was found that microglia play a significant role in Arf1‐deficiency induced neurodegeneration and knocking out NLRP3 can effectively rescue this neurodegenerative phenomenon. Consistently, other researches also identified the activation of microglia contributed to various neurodegenerative diseases.^[^ ^44^, ^45^, ^46^ ^]^ Based on these results, we here pursued how the Arf1‐deficiency mediated lipid dysregulation and microglia activation. Detailed mechanism studies showed that the accumulation of lipid droplets and oxidized lipids, which, upon transference to microglia, activated the NLRP3 inflammasome, thereby facilitating the production of IL‐1β.^[^ ^16^ ^]^ The IL‐1β, along with chemokines, recruited and activated γδT cells in the meninges, and the INF‐γ released from γδT cells activated a microglia‐A1 astrocyte‐C3‐neuron C3aR circuit.^[^ ^17^ ^]^ Knockout of NLRP3, IFN‐γ or C3 significantly rescued the neurodegenerative phenotypes caused by Arf1 gene knockout.^[^ ^16^, ^17^ ^]^ In this study, we further elucidated a detailed molecular mechanism (Figure S9C, Supporting Information). We found that Arf1‐deficiency first induced surplus fatty acid synthesis through the AKT‐mTORC1‐SREBP1‐FASN axis, which further triggered endoplasmic reticulum (ER)‐mitochondrial stress cascade via calcium flux. The organelle stress cascade further caused mtDNA to be released into cytoplasm. Meanwhile, the FASN‐driven fatty acid synthesis in the Arf1‐deficient neurons might also induce accumulation of sphingolipids in lysosomes that caused dysfunction of autophagy and lysosomes,^[^ ^29^ ^]^ which was associated with lysosomal stress and MDEV release,^[^ ^47^ ^]^ the released EVs carried mtDNA into microglia to activate the inflammatory (cGAS‐STING and NLRP3 inflammasome) pathways to promote neurodegeneration. The increased ROS and MDEV release might also promote formation and release the above mentioned oxidized lipids.^[^ ^16^, ^17^ ^]^

Moreover, in the mouse model, both knockdown of Fasn at the genetic level and reducing accumulation of sphingolipids in lysosomes with Myriocin treatment in vivo significantly rescued neurodegenerative phenotypes of the Arf1‐deficient mice. The *de novo* synthesis of lipids necessitates a series of enzymatic reactions. Acetyl‐CoA, under the influence of Acetyl‐CoA Carboxylase (ACC), forms malonyl‐CoA, a crucial substrate for fatty acid synthesis. Subsequently, seven molecules of malonyl‐CoA and one molecule of acetyl‐CoA undergo a series of cyclic reactions catalyzed by Fatty Acid Synthase (FASN), culminating in the synthesis of palmitic acid.^[^ ^48^, ^49^, ^50^ ^]^ This study emphasizes the role of initial lipid synthesis in neurodegenerative diseases but does not delve into subsequent, more detailed aspects of lipid metabolism, such as the length of lipid carbon chains, the saturation of lipids, phospholipid metabolism, and cholesterol metabolism. Although our research found that blocking the FASN mediated lipid synthesis can to some extent rescue neurodegeneration caused by Arf1 deficiency, the potential function of redundancy pathway has not been fully exclusive and the specific lipid molecules that exert biological functions downstream still require further investigation. For instance, it has been discovered that long‐chain saturated fatty acids secreted by activated astrocytes performed neurotoxicity by inducing endoplasmic reticulum stress.^[^ ^51^ ^]^ The lipid composition within cells is complex and diverse, further experiment will be needed to systematically illustrate the interaction between the lipid metabolism pathway.

Both the microglial LDs (LDAM, identified in the Wyss‐Coray's laboratory)^[^ ^9^, ^10^ ^]^ and the neuronal LDs (identified in our laboratory)^[^ ^16^, ^17^ ^]^ merge on activation of inflammatory (cGAS‐STING and NLRP3 inflammasome) pathways in microglia. However, the cytokine (possibly similar to IL‐1β) released from LDAM was demonstrated to directly promote Tau phosphorylation and neurotoxicity based on cell culture experiment.^[^ ^10^ ^]^ The IL‐1β released from inflammatory microglia activated by the neuronal LDs in the Arf1‐ablated neurons was demonstrated to trigger neurodegeneration through activating a long neuron‐immune and microglia‐A1 astrocyte‐C3‐neuron C3aR circuit based on our studies.^[^ ^16^, ^17^ ^]^ Further studies are required to understand better whether a direct or a long circuit or both mechanisms mediate the pathological processes of LDs in various central nervous system cell types.

### 3.2 Relationship Between Arf1 and ND Models

Amyotrophic lateral sclerosis (ALS) is a neurodegenerative disease characterized by impaired motor function, primarily affecting motor neurons in the brain and spinal cord. The etiology of ALS remains unclear. To date, over 30 genes have been identified as being associated with the development and progression of ALS, among which mutations in C9orf72, SOD1, TARDBP, and FUS account for ≈70% of familial ALS cases.^[^ ^52^ ^]^ Interestingly, the complex formed by C9orf72, SMCR8, and WDR41 possesses GTPase‐activating protein (GAP) activity and is capable of interacting with ARF1 to catalyze the conversion of active ARF1‐GTP to inactive ARF1‐GDP, thereby regulating the activity of ARF1.^[^ ^53^ ^]^ In addition to these genetic factors, the clinical manifestations of ALS include motor dysfunction, accompanied by robust oxidative stress, activation of neuroinflammation, mitochondrial dysfunction, and DNA damage.^[^ ^52^ ^]^ These findings suggest that in ALS patients, particularly those with C9orf72 mutations, ARF1‐mediated lipid metabolism may play a significant role in the disease pathology.

Additionally, as previously mentioned, the R1819W mutation in FASN can lead to cognitive impairments in patients who are diagnosed with AD.^[^ ^23^ ^]^ Furthermore, in another study on AD patients, we found that some patients carry the V2005A point mutation in FASN.^[^ ^35^, ^54^ ^]^ Although the significance of this mutation is not high due to the limited cohort size, it also suggests the possibility that FASN point mutations can cause cognitive impairments. We also plan to construct a mouse model carrying the V2005A point mutation in FASN to further investigate the role of FASN point mutations in neurodegenerative diseases. A large‐scale population cohort study on PD conducted recently identified a synonymous mutation in FASN, A1089A, in PD patients.^[^ ^37^ ^]^ We hypothesize that with the expansion of population cohorts, more mutations in FASN may be discovered in these neurodegenerative patients.

In summary, the lipid metabolic disorder mediated by FASN resulting from Arf1 deficiency is likely to play a significant role in the aforementioned ND patients. We anticipate that similar phenomena will be observed and further elucidated in patients and mouse models of ALS, AD, and PD in future studies.

### 3.3 Neuron Spreads the Inflammatory Factors to Neighboring Cells Through Releasing mtDNA via MDEVs

A multitude of studies have underscored the DNA‐sensing cGAS‐STING pathway as a pivotal neuroinflammatory axis, integral to the pathogenesis of a spectrum of neurodegenerative diseases, such as PD, HD, AD, and ALS.^[^ ^55^ ^]^ The nucleus‐derived cytoplasmic formation of chromatin fragments (CCFs) and activation of retrotransposons could activate the DNA‐sensing cGAS–STING pathway.^[^ ^56^ ^]^ Here we demonstrated that damaged mtDNA triggered by disorders of lipid metabolism, not genomic DNA, activated this pathway, mediating the crosstalk between neurons and microglia in NDs. Previous publications have demonstrated intercellular transfer of mitochondria components and mtDNA, which could spread the inflammatory factors to neighboring cells^[^ ^57^, ^58^, ^59^ ^]^; however, the transfer mechanism of these factors between cells remain to be determined.  By specific mtDNA labeling and EV purification, we illustrated that the CD63 positive‐EVs participated in intercellular mtDNA transfer. Importantly, both mtDNA clearance and EV elimination could effectively reverse neuroinflammation. To rule out the possibility of classical exosomes, we employed GW4869,^[^ ^60^ ^]^ a well‐established inhibitor of exosome releasing, to inhibit exosome release in N2a cells. Surprisingly, the use of GW4869 led to a significant increase in mitochondrial components (Figure S11A, Supporting Information) and mtDNA components in the cell culture supernatant (Figure S11B‐C, Supporting Information). This result suggested that the MDEVs we observed may not be secreted extracellularly in a classical exosomal manner. As expected, the GW4869 treatment could not rescue neuron damage of Arf1‐KO mice model (Figure S11D‐H, Supporting Information). The detection of these components in the purified exosome fractions may be attributed to the similar sizes of MDEVs and exosomes, which prevents their separation during the PEG precipitation purification process of EVs. However, we cannot exclude the possibility that some mtDNA may be secreted to outside through cell membrane ruptures, intercellular connection channels, migrasome, or other alternative pathways.

In this study, we discovered that mtDNA is secreted in the form of MDEVs into microglial cells. Therefore, we anticipated that the classic exosome inhibitor GW4869 could inhibit the release of MDEV and thus block the transfer of mtDNA. However, treatment of mice or N2a cells with GW4869 failed to rescue the neurodegenerative phenotype or inhibit MDEV release (Figure S11, Supporting Information), suggesting that MDEV was secreted via a novel pathway. We plan to meticulously isolate MDEVs from mixed EV preparations, characterize their composition via LC‐MS, and further investigate the mechanisms mediating MDEV release.

We hypothesize that lysosomal dysfunction triggered by abnormal sphingolipid metabolism plays a crucial role in the release of MDEV based on our preliminary data. To further validate this hypothesis, we plan to construct mouse models of Thy1‐CreER; Arf1^f/f^; Sgms1^f/f^ and Thy1‐Cre; Arf1^−/−^; Sptlc1^−/−^ to confirm whether blocking sphingolipid synthesis can effectively rescue neurodegeneration caused by Arf1 deficiency. Additionally, we aim to explore the molecular mechanisms by which abnormal sphingolipid metabolism regulates MDEV release.

We want to identify key proteins involved in MDEV release and elucidate the components of MDEV. By knocking out these key proteins or intracranially injecting corresponding antibodies to neutralize MDEV, we hope to inhibit the release of MDEV and thereby prevent the transfer of mtDNA.

**Table 1:** Key Resources Table.

REAGENTS or RESOURCE	SOURCE	IDENTIFIER
**Antibodies**		
anti‐Beta Actin antibody	Proteintech	Cat# 81115‐1‐RR; RRID: AB_2923704
anti‐ARF1 antibody	ABclonal	Cat # A9195; RRID: AB_2923704
anti‐p‐PERK antibody	Cell Signaling Technology	Cat# 3179; RRID: AB_2095853;
anti‐BIP antibody	Cell Signaling Technology	Cat# 3179; RRID: AB_2095853;
anti‐ATF4 antibody	Cell Signaling Technology	Cat# 11815; RRID: AB_2616025;
anti‐p‐AKT antibody	Proteintech	Cat# 66444‐1‐Ig; RRID: AB_2782958;
anti‐AKT antibody	Proteintech	Cat# 60203‐2‐Ig; RRID: AB_10912803;
anti‐p‐S6K antibody	Cell Signaling Technology	Cat# 9234; RRID: AB_2269803;
anti‐S6K antibody	Proteintech	Cat# 14485‐1‐AP; RRID: AB_2269787;
anti‐SREBP1 antibody	Abcam	Cat# ab28481; RRID: AB_778069;
anti‐FASN antibody	Proteintech	Cat# 10624‐2‐AP; RRID: AB_2100801;
anti‐p‐TBK1 antibody	Cell Signaling Technology	Cat# 5483; RRID: AB_10693472;
anti‐TBK1 antibody	Proteintech	Cat# 28397‐1‐AP; RRID: AB_2881132;
anti‐cGAS antibody	Santa Cruz	Cat# sc‐515777; RRID: AB_2734736;
anti‐p‐STING antibody	Affinity	Cat# AF7416; RRID: AB_2843856;
anti‐STING antibody	Proteintech	Cat# 19851‐1‐AP; RRID: AB_10665370;
anti‐HSP60 antibody	Proteintech	Cat# 15282‐1‐AP; RRID: AB_2121440;
anti‐ATP5A antibody	Abcam	Cat# ab14748; RRID: AB_301447;
anti‐Tomm20 antibody	Proteintech	Cat# 11802‐1‐AP; RRID: AB_2207530;
anti‐CD63 antibody	Santa Cruz	Cat# sc‐5275; RRID: AB_627877;
anti‐CD9 antibody	Proteintech	Cat# 20597‐1‐AP; RRID: AB_2878706;
anti‐CD81 antibody	Proteintech	Cat# 66866‐1‐Ig; RRID: AB_2616025;
anti‐NLRP3 antibody	Cell Signaling Technology	Cat# 15101; RRID: AB_2722591;
anti‐p‐p65 antibody	Cell Signaling Technology	Cat# 11815; RRID: AB_331284;
anti‐p65 antibody	Cell Signaling Technology	Cat# 8242; RRID: AB_10859369;
anti‐IL‐1 beta antibody	GnenTex	Cat# 11815; RRID: AB_378141;
anti‐Caspase 1/p20/p10 antibody	Proteintech	Cat# 22915‐1‐AP; RRID: AB_2876874;
anti‐DNA antibody	Sigma	Cat# CBL186; RRID: AB_11213573;
Goat Anti‐Rabbit IgG H&L‐Alexa Fluor 488	Abcam	Cat# ab150077; RRID: AB_2630356;
Goat Anti‐Rabbit IgG H&L‐Cy3	Abcam	Cat# ab6939; RRID: AB_955021;
Goat Anti‐Rabbit IgG H&L‐Alexa Fluor 647	Abcam	Cat# ab150079; RRID: AB_2722623;
Goat Anti‐Mouse IgG H&L‐Alexa Fluor 488	Abcam	Cat# ab150113; RRID: AB_2576208;
Goat Anti‐Mouse IgG H&L‐Cy3	Abcam	Cat# ab97035; RRID: AB_10680176;
Goat Anti‐Mouse IgG H&L‐Alexa Fluor 647	Abcam	Cat# ab150115; RRID: AB_2687948;
anti‐MAP2 antibody	Proteintech	Cat# 67015‐1‐Ig; RRID: AB_2882331;
anti‐NeuN antibody	Proteintech	Cat#2 6975‐1‐AP; RRID: AB_2880708;
anti‐IBA1 antibody	Proteintech	Cat# 10904‐1‐AP; RRID: AB_2224377;
anti‐GFAP antibody	Proteintech	Cat# 60190‐1‐Ig; RRID: AB_10838694;
anti‐LC3 antibody	Proteintech	Cat# 14600‐1‐AP; RRID: AB_2137737;
anti‐p62 antibody	Proteintech	Cat# 18420‐1‐AP; RRID: AB_10694431;
**Chemicals, peptides, and recombinant proteins**		
Rapamycin	Selleck	Cat# S1039
Fatostatin HBr	Selleck	Cat# S8284
TVB‐3664	Selleck	Cat# S8563
MCU‐i4	Selleck	Cat# S9842
CsA	Selleck	Cat# S2286
Zalcitabine	Selleck	Cat# S1719
Tamoxifen	Selleck	Cat# S1238
GCA	Selleck	Cat# S7266
Myriocin	MedChemExpress	Cat# HY‐N6798
BODIPY™ 493/503	Thermo Fosher	Cat# D3922
BODIPY™ 500/510 C1, C12	Thermo Fosher	Cat# D3823
TMRE	Thermo Fosher	Cat# T669
Hoechst	Thermo Fisher	Cat# H1399
Rhod‐2, AM	Yeasen	Cat# 40776
UCF.ME UltraNuclease	Yeasen	Cat# 20156
0.5M EDTA, pH8.0 (Sterile, Cell Culture Grade)	Beyotime	Cat# C0196
BeyoMag™ Streptavidin Magnetic Beads	Beyotime	Cat# P2151
DAPI	Sigma	Cat# D9542
Trizol	Beyotime	Cat# R0016
NovoScriptPlus All‐in‐one 1st Strand cDNA Synthesis SuperMix	Novoprotein	Cat# E047
DMEM	BasalMedia	Cat# L110KJ
Fetal Bovine Serum (FBS)	Lonsera	Cat# s711‐001s
Salt Active UltraNuclease GMP‐grade	Yeasen	Cat# 20159ES25
Amicon Ultra filter, 100 kDa MWCO	Sigma	Cat# UFC9100
0.45µm PVDF membrane	Sigma	Cat# IPVH00010
**Critical commercial assays**		
ROS Assay Kit	Beyotime	Cat# S0033M
MPTP Assay Kit	Beyotime	Cat# C2009S
Exosome Extraction Kit	Beyotime	Cat# C3620M
NP‐40 Lysis Buffer	Beyotime	Cat# P0013F
RIPA Lysis Buffer	Beyotime	Cat# P0013B
8‐OHdG ELISA Ki	Elabscience	Cat#E‐EL‐0028
Circulating DNA extraction kit	Onrew	Cat# DNC614
Exosome Isolation Kit from Cell Culture Media by Precipitation Method	Beyotime	Cat# C3620S
Universal Virus Concentration Kit	Beyotime	Cat# C2901M
**Deposited data**		
RNA‐seq	GSE273284	
**Experimental models: Cell lines**		
HEK 239T/17	ATCC	Cat# CRL‐11268™
Neuro‐2a	ATCC	Cat# CCL‐131
Eoc20	ATCC	Cat# CRL‐2469
**Experimental models**:		
**Organisms/strains**		
Mouse: Thy1‐CreER (SLICK‐H)	The Jackson Laboratory	Cat# 012708
**Recombinant DNA**		
pLKO.1‐shLuciferase‐puromycin	This paper	N/A
pLKO.1‐shArf1‐1‐puromycin	This paper	N/A
pLKO.1‐shArf1‐2‐puromycin	This paper	N/A
pcDNA3‐Tomm20‐mCherry	This paper	N/A
psPAX2	Addgene	Cat# 12260
pMD2.G	Addgene	Cat# 12259
pHelper	Provided by Fuqiang Xu	N/A
pUCmini‐iCAP‐PHP.eB	Provided by Fuqiang Xu	Cat# 103005
pAAV‐hSyn‐EGFP	This paper	N/A
pAAV‐hSyn‐LIPM	This paper	N/A
pAAV‐U6‐shLuciferase	This paper	N/A
pAAV‐U6‐shFasn	This paper	N/A
pLX304 CMV OMM‐FKBP‐V5‐sTurboID (N)	Addgene	Cat# 153006
pLX208 CMV sturboID (C)‐HA‐FRB	Addgene	Cat# 153007
pCMV‐N‐mito‐Flag‐APEX2	Beyotime	Cat# D3047
**Software and algorithms**		
FlowJo_V10	Becton Dickinson	https://www.flowjo.com/solutions/flowjo
GraphPad Prism10	GraphPad	https://www.graphpad.com/
Image J	Image J	https://imagej.net/software/imagej/
BioRender	N/A	https://www.biorender.com/

Based on our results, we hypothesize that in the absence of Arf1, there may be an induction of massive lipid synthesis, wherein FASN‐generated palmitic acid can serve as a precursor for sphingolipid synthesis, thereby further triggering a substantial production of sphingolipids. The accumulation of lipids within the cell may disrupt the calcium homeostasis between the ER‐mitochondria, leading to the accumulation of calcium ions in the mitochondria and the subsequent opening of the mitochondrial permeability transition pore (mPTP), which results in mitochondrial damage. Under normal conditions, damaged mitochondria are rapidly degraded through autophagy. However, due to the substantial accumulation of sphingolipids in lysosomes, which impairs their degradative function, damaged mitochondria within autophagosomes cannot be degraded upon fusion with lysosome. In such cases, cells may fuse these undigested autophagosomes with the plasma membrane, thereby releasing the damaged mitochondria in the form of extracellular vesicles. Thus, our studies suggested that the neuronal mtDNA triggered by disorders of lipid metabolism is the pathogenic factor of neuroinflammation. Considering that abnormal EVs are discovered in multiple ND diseases,^[^ ^61^, ^62^, ^63^ ^]^ the mechanism presented in this study may also provide novel diagnostic and therapeutic approaches to the diseases.

## 4 Experimental Section

### Experimental Model and Subject Details

*Mice*: All animals were maintained under specific pathogen‐free (SPF) conditions at the Laboratory Animal Center of the Institute of Developmental Biology and Molecular Medicine, Fudan University. All animal experiments followed the animal study protocols approved by the Animal Care and Use Committee of Fudan University. The Arf1‐floxed mice were generated in the animal core facility of the Mouse Cancer Genetics Program at NCI, as described previously.^[^ ^64^ ^]^ The Thy1‐CreERT2 mice were obtained from The Jackson Laboratory.

*Cells*: Neuro‐2a, Eoc20, and HEK 293T/17 cells were cultured in DMEM (BasalMedia L110KJ) containing 10% FBS and 1% penicillin/streptomycin. All cells were grown in an atmosphere containing 5% CO2 at 37 °C. The detailed materials were shown in **Table** **1**.

### Method Details

*Primary Neuron and Microglia Culture*: Primary culture of mouse neurons was performed as described previously.^[^ ^65^ ^]^ Pregnant female mice with E17.5.5 embryos were anesthetized and embryos were isolated from the uterus. The hindbrain was dissected, placed in ice‐cold HBSS, and digested with papain solution at 37 °C for 30 min. Brain lysates were centrifuged for 5 min at 800 r.p.m. at room temperature. The cell pellet was resuspended in Hank's solution containing DNase I and then dissociated into single cells by gentle pipetting up and down using a 1 mL pipette. After that, cells were transferred into Hank's solution containing 10 mg mL^−1^ trypsin inhibitor (Sigma, T9253) and 10 mg mL^−1^ BSA (Sigma, A9647). Cells were centrifuged at 800 r.p.m. for 10 min and resuspended in neurobasal medium (2% fetal bovine serum (Gibco, 10437028), 2% B27 supplements (Thermo Fisher, 17504044)) and 2 mM L‐glutamine (Thermo Fisher, 25030149). Neurons at a density of 0.8.9 × 105 cells per well were plated on 12‐mm glass coverslips residing in 24‐well plates that were coated with poly‐d‐lysine (Sigma, P6407). The neuron medium was changed by a half volume once a week, and included 2% B27 neurobasal maintenance medium (GIBCO, 17504044) supplements and 2 mM L‐glutamine.

The details of primary culture of mouse brain microglia cells were described previously.^[^ ^66^ ^]^ The P1 pups were killed by CO_2_; their heads were decapitated using an autoclaved scissor and placed into a 6‐cm Petri dish containing 6 mL cold dissection medium. The pup skull was opened using a scissor and the brain was isolated. The meninges were carefully removed, and the hindbrain and brain stem were collected and washed with dissection medium (1 × HBSS, 10 mM HEPES, 6 mg glucose powder, and 100 U mL^−1^ Pen/Strep solution) once. Tissues were digested with 30 mL dissection medium containing 1.5 mL 2.5% trypsin for 15 min in an incubator at 37 °C, followed by addition of 1.2 mL of 1 mg mL^−1^ trypsin inhibitor and incubating for 1 min and adding 750 µL of 10 mg mL^−1^ DNase to digest the sticky DNA. dissection cells were centrifuged at 400g for 5 min and the cell pellet was resuspended into 5 mL warmed culture medium (DMEM + 10% FBS). Each well of A 24‐well plate was seeded with 60 000 cells and cultured in a cell culture incubator with 5% CO2, 100% humidity at 37 °C.

*Lentiviral Synthesis and Transduction*: Lentiviral particles were made by polyethyleneimine (concentration was 1 mg mL^−1^) ‐based co‐transfection of HEK297T cells with the targeted plasmids and the packaging plasmids psPAX2 and pMD2.G in a ratio of 4:3:2. Virus‐containing media was collected 48 h after transfection and then centrifuged at 4400 rpm for 5 mins, collect supernatant.

*Immunoblot Analyses*: For immunoblotting, cells were lysed for total protein using 1 × loading cell lysis buffer, followed by incubation in a 95 °C‐metal bath for 15 min. The samples were separated on 8%‐15% SDS‐PAGE gel and transferred to PVDF membrane (Cat#IPVH00010, Millipore). At room temperature, the membrane was blocked in 5% skim milk powder for at least 1h and then incubated in the primary antibody at 4 °C overnight. TBST was then used to clean the film thrice for 10 min each, then incubated with the HRP‐conjugated secondary antibody at room temperature for 1h. After washing again, chemiluminescence imaging was performed using an ECL luminescent solution.

*Quantitative Reverse‐Transcription PCR*: RNAs were extracted from cells using Trizol (Beyotim), and complemental DNA was generated using NovoScriptPlus All‐in‐one 1st Strand cDNA Synthesis SuperMix (Cat. No.:E047, Novoprotein) according to the manufacturer's instructions. Real‐time PCR was performed using the 2 × Trans‐Start Top Green qPCR SuperMix (Transgene). The PCR results were normalized to Actin expression.

*Immunofluorescence (IF) Analyses;* N2a or Eoc20 cells were placed on the cell sheet two days before. After 24h of treatment with related drugs, the cells were fixed with 4%PFA for 20 min, cleaned with PBS three times for 10 min each time, and then permeated with PBST, which contained 0.1% TritonX‐100, for 15 min, followed by blocking with 10% FBS for 1h and then incubated with the primary antibodies at 4 °C overnight. Slides were then washed with PBS three times for 10 min each time and incubated with the secondary antibody for 1h at room temperature. After using DAPI to label nuclei, the slides were rewashed with PBS and sealed with a mounting medium.

*Lipid Uptake and Secretion Assay*: Cells were pre‐cultured with BODIPY C1, C12, a fatty acid analog, contained medium for 30 min and then replaced with fresh medium. A portion of these cells were collected for flow cytometry to analyze differences among these cells' fatty acid uptake. Another portion of the cells was collected after another 8 h of culture for flow cytometry to analyze the differences in fatty acid secretion. The last part of the cells was collected for flow cytometry after another 24 h of culture to further investigate the cells' lipid secretion and ability to retake lipids.

*Cytosolic and Supernatant DNA Extraction*: The cells were digested in the six‐well plate to extract cytosolic DNA, and 1/5 of the cell was taken to extract total RNA. The equivalent volume of RNA was reverse‐transcribed for subsequent qPCR experiments. The remaining 4/5 cell samples were incubated with 1ml of plasma membrane lysate (containing 150 mM of sodium chloride, 50 mM of HEPES, and 25 µg mL^−1^ of digitonin) on a turntable at 4 °C for 10 min to permeate the plasma membrane. The cell lysate was centrifuged at 4 °C at a speed of 13 800 rpm for 10 min. At this time, the supernatant was pure cytoplasm, and the precipitation was the nucleus and other organelle components. The supernatant was carefully absorbed into a clean 1.5 mL centrifuge tube. Then, 50 µL RNase A (concentration of 10 mg mL^−1^) was incubated at room temperature for 10 min to remove RNA from the cytoplasm. Then, DNA was extracted from the cytoplasm using the Circulating DNA Extraction Kit.

For supernatant DNA extraction, the collected cell culture medium was centrifuged at 4400 rpm for 5 min to remove cell debris, the centrifuged supernatant was carefully transferred to a clean centrifuge tube, and then DNA was extracted from the supernatant using Circulating DNA Extraction Kit. The cultured cells were collected to extract total RNA, which was reverse‐transcribed for subsequent qPCR experiments.

*Supernatant Protein Extraction*: First, cultured cells are collected as inputs for cell quantification. Subsequently, the collected cell culture medium was centrifuged at 4400 rpm for 5 min to remove cell debris. 500 µL of the centrifuged supernatant was carefully absorbed and transferred to a clean centrifuge tube, where 500 µL methanol and 125 µL trichloromethane were added. After vortexing the mixture, centrifuge at 4 °C at 13 000 rpm for 5 min. Discard the upper phase, add 500 µL methanol, and centrifuge at 4 °C at 13 000 rpm for 5 min. Remove supernatant carefully, dry in 50 °C metal for 5 min, and add 1 × loading to dissolve protein.

*Exosome Extraction*: For exosome purification, for each sample, 2 trays of 10 cm dishes each were prepared in advance, and cells were cultured for 24 h using serum‐free DMEM. The cultured cells were collected for cell counting, and equal amounts of cells were collected and scaled to collect cell culture medium. The collected medium was first centrifuged at 4400 rpm for 5 min at room temperature to remove larger cellular debris initially; the supernatant was carefully aspirated and transferred to a clean centrifuge tube, followed by centrifugation at 15 000 g for 1 h at 4 °C to remove smaller cellular debris and more giant extracellular vesicles. The centrifuged supernatant was then transferred to an Amicon Ultra filter (100 kDa MWCO) and centrifuged at 4000 rpm for 10 min at 4 °C to initially concentrate the exosomes, followed by resuspension of the concentrate with 800 µL of PBS. The resuspension will be used for subsequent exosome purification according to the instructions of the Cell Serum Exosome Extraction Kit (Precipitation Method).

*Nanoparticles Tracking Analysis;* ZetaView PMX 110 was used for nanoparticle tracking analysis of purified exosomes. The sample pool was first cleaned with deionized water, followed by calibration of the instrument with a polystyrene microsphere (100 nm) standard, and then the sample pool was cleaned three times with PBS. Afterwards, the purified exosomes were diluted 4000 times with PBS, and then the diluted exosomes were injected into the sample pool for detection. The acquired data were then analyzed using ZetaView 8.04.02 SP2.

*Detection of mtDNA in MDEVs*: Pre‐incubating the cells with SG‐BR for 30 min, followed by treatment with DMSO or GCA (an Arf1 inhibitor) for 24 h. Subsequently, the cells were cultured for an additional 24 h in serum‐free medium. EVs were then isolated and purified from the cell culture supernatant, and the abundance of mtDNA within these EVs was analyzed by flow cytometry, specifically by detecting the B615 channel. The flow cytometer utilized was the CytoFLEX LX from Beckman, which is capable of analyzing nanoparticles with a minimum detection limit of 80 nm.

*AAV Production and Quantification*: AAV‐PHP.eB expressing shRNA was produced and purified as described in the official protocol for Addgene. In brief, pAAV‐U6‐shLuciferase or pAAV‐U6‐shFasn and AAV packaging plasmids (pUCmini‐iCAP‐PHP.eB and pHelper) were co‐transfected into HEK 293T cells using polyethyleneimine at the ratio of 125:248:182 based on micrograms of DNA with 111 µg in total per 150‐mm dish. 72 h after transfection, viral particles were collected from the medium and cells. The mixture of cells and medium was centrifuged to form cell pellets. The cell pellets were suspended in 500 mM NaCl, 40 mM Tris, 10 mM MgCl2, pH ≈10, and 100 U mL^−1^ of Salt Active UltraNuclease at 37 °C for 1 h. The mixture was finally subjected to subsequent purification by using the Universal Virus Concentration Kit. Virus titers were determined using quantitative PCR to measure the number of viral genomes (vg) after DNase I treatment to remove the DNA not packaged and then proteinase K treatment to digest the viral capsid and expose the viral genome. Quantified plasmids of pAAV‐U6‐shLuciferase or pAAV‐U6‐shFasn were used as a DNA standard to transform the Ct value to the amount of the viral genome.

*Mice Behavioral Experiments*: After tail vein injection of AAV, mice were bred for 14 days for shRNA expression or 21 days for target gene expression, followed by five consecutive days of intraperitoneal injection of tamoxifen (at a dose of 100 mg kg^−1^) to induce knockdown of Arf1. Subsequently, the mice's body weights were monitored daily and compared with the first day's. During this period, mice underwent a treadmill, gait, and open‐field experiments. For the treadmill experiment, mice will undergo a week‐long treadmill exercise before the formal experiment, exercising at 5 m min^−1^, 10 m min^−1^, and 15 m min^−1^ for 3 min During the formal experiment, mice will perform the treadmill experiment every day, and mice that are shocked more than 15 times in 15 s will be considered exhausted. For the gait experiment, mice will be subjected to a gait experiment every two days, and gait will be analyzed at a speed of 10 cm s^−1^. For the open field experiment, mice will be subjected to an open field experiment every five days, and each mouse will move in the open field for 10 min. The mice will be analyzed in terms of the time they stay in the different areas and the distances traveled.

## Conflict of Interest

The authors declare no conflict of interest.

## Author Contributions

X.L., Y.W., and S.X.H. conceived and designed the experiments. X.L., Y.W., D.W., S.J., L.S., X.T., Y.L., and T.Y. performed the experiments and analyzed the data. S.H. provided technology support. X. L., Y. W., and S.X.H. wrote the manuscript.

## Supporting information



Supporting Information

Supplemental Video 1

Supplemental Video 2

## Data Availability

The data that support the findings of this study are available on request from the corresponding author. The data are not publicly available due to privacy or ethical restrictions.
