# Supplementary material for: Accumulation of Damaging Lipids in the Arf1‐Ablated Neurons Promotes Neurodegeneration through Releasing mtDNA and Activating Inflammatory Pathways in Microglia
Source: Adv Sci (Weinh). 2025 Feb 28;12(16):2414260. doi: 10.1002/advs.202414260 (PMC12021055; doi:10.1002/advs.202414260)
Supplement: Supplementary file 1 — Supporting Information [file ADVS-12-2414260-s003.docx]

**Supplemental file for**

**Accumulation of damaging lipids in the Arf1-ablated neurons promotes neurodegeneration through releasing mtDNA and activating inflammatory pathways in microglia**

**Xu Li^1^, Shuhan Jin^1^, Danke Wang^1^, Ying Wu^1^, Xiaoyu Tang^1^, Yufan Liu^1^, Tiange Yao^1^, Shoufa Han^2^, Lin Sun^1^, Yuetong Wang^1,^ *, Steven X. Hou^1,^ ***

^1^Department of Cell and Developmental Biology at School of Life Sciences, State Key Laboratory of Genetic Engineering, Institute of Metabolism and Integrative Biology, Children’s Hospital, Zhongshan Hospital, Fudan University, Shanghai 200438, China

^2^State Key Laboratory for Physical Chemistry of Solid Surfaces, Department of Chemical Biology, College of Chemistry and Chemical Engineering, The Key Laboratory for Chemical Biology of Fujian Province, The MOE Key Laboratory of Spectrochemical Analysis & Instrumentation, Innovation Center for Cell Signalling Network, Xiamen University, Xiamen, 361005, China.

*Correspondence: stevenhou@fudan.edu.cn (S.X.H.)

wangyt@fudan.edu.cn (Y.T.W)

Keywords: Arf1 ablation, surplus fatty acid synthesis, mtDNA release, neuron degeneration

**
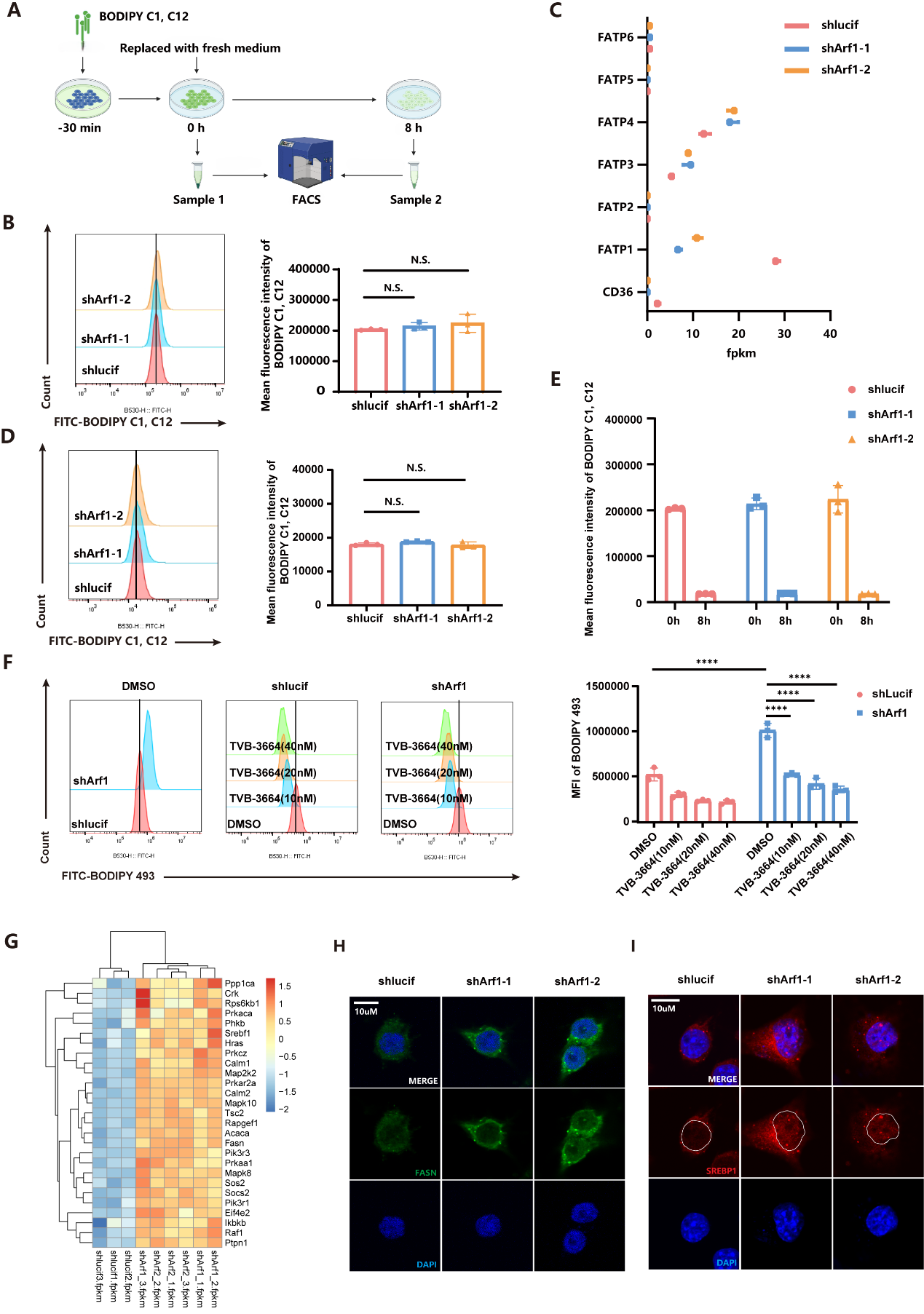
Supplemental Figure 1. Arf1 deficiency enhanced lipid de novo synthesis by activating the mTORC1-SREBP1-FASN axis to trigger lipid accumulation, related to Figure 1.**

1. Schematic of the lipid uptake and secretion assay.
2. Flow cytometry of BODIPY C1, C12 from control or Arf1-deficient N2a cells at 0h. The representative FACS plots and mean fluorescence intensity of BODIPY C1, C12 are shown. n = 3 per group.
3. Relative expression of lipid transporters of RNA-seq obtained from control or Arf1-deficient N2a cells. n = 3 per group.
4. Flow cytometry of BODIPY C1, C12 from control or Arf1-deficient N2a cells at 8h. The representative FACS plots and mean fluorescence intensity of BODIPY C1, C12 are shown. n = 3 per group.
5. The comparison of the mean fluorescence intensity of BODIPY C1, C12 from control or Arf1-deficient N2a cells between 0h and 8h. n = 3 per group.
6. Flow cytometry of BODIPY 493 from control or Arf1-deficient N2a cells treated with gradient dose of TVB-3664. The representative FACS plots and mean fluorescence intensity of BODIPY 493 are shown. n = 3 per group.
7. Heatmap analysis of RNA-seq obtained from control and Arf1-deficient N2a cells. n = 3 per group.
8. Representative FASN images of control and Arf1-deficient N2a cells.
9. Representative SREBP1 images of control and Arf1-deficient N2a cells.

Data are shown as the mean ± SEM. *P < 0.05, **P < 0.01. ***P < 0.001. ****P < 0.0001 by two-way ANOVA with Holm Šídák’s multiple comparisons test.

**
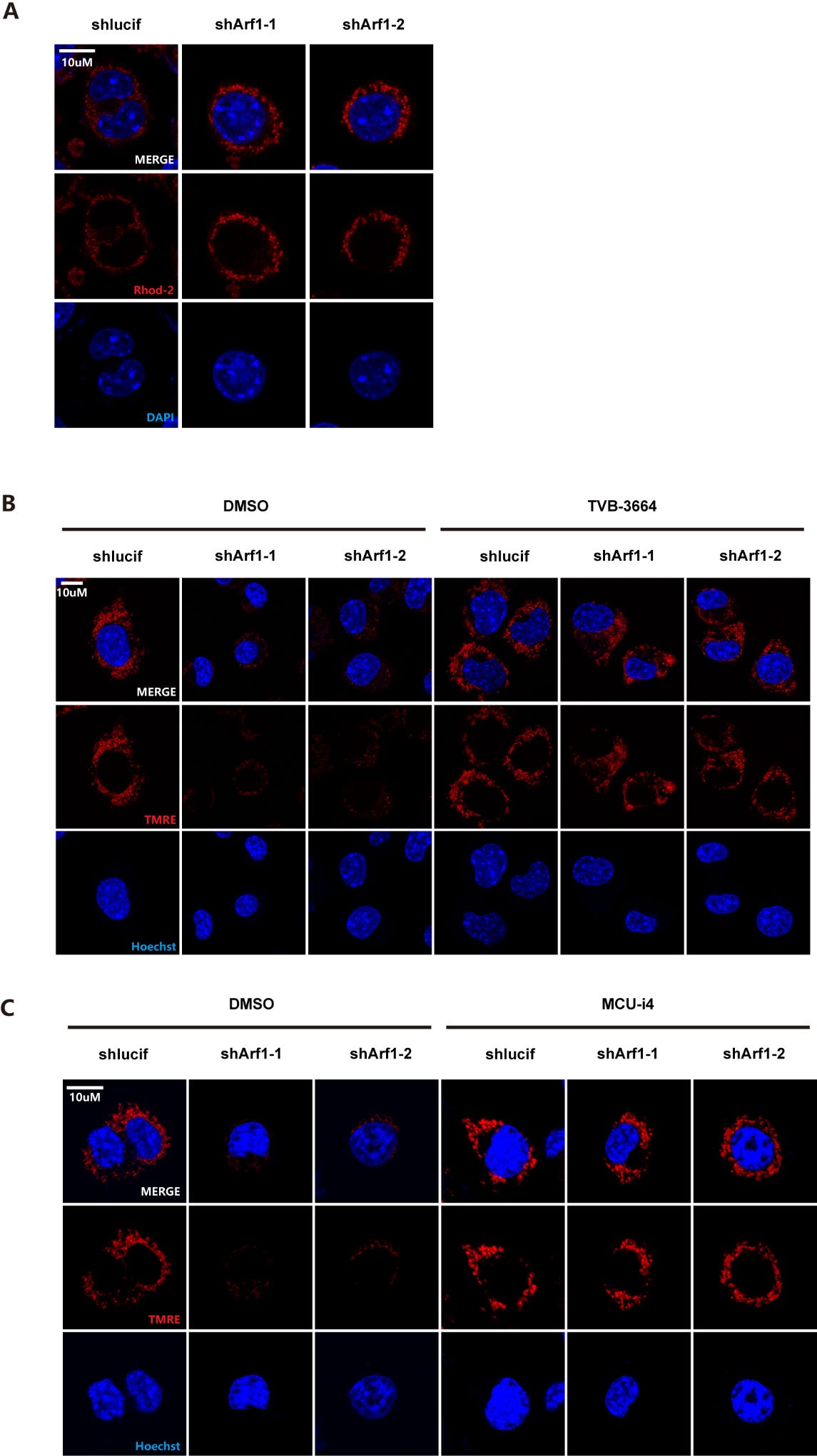
Supplemental Figure 2. Elevated lipid levels triggered the stress cascade of ER-mitochondria organelles, related to Figure 2.**

1. Representative Rhod-2 images of control or Arf1-deficient N2a cells.
2. Representative TMRE images of control or Arf1-deficient N2a cells treated with DMSO or TVB-3664.
3. Representative TMRE images of control or Arf1-deficient N2a cells treated with DMSO or MCU-i4.

**
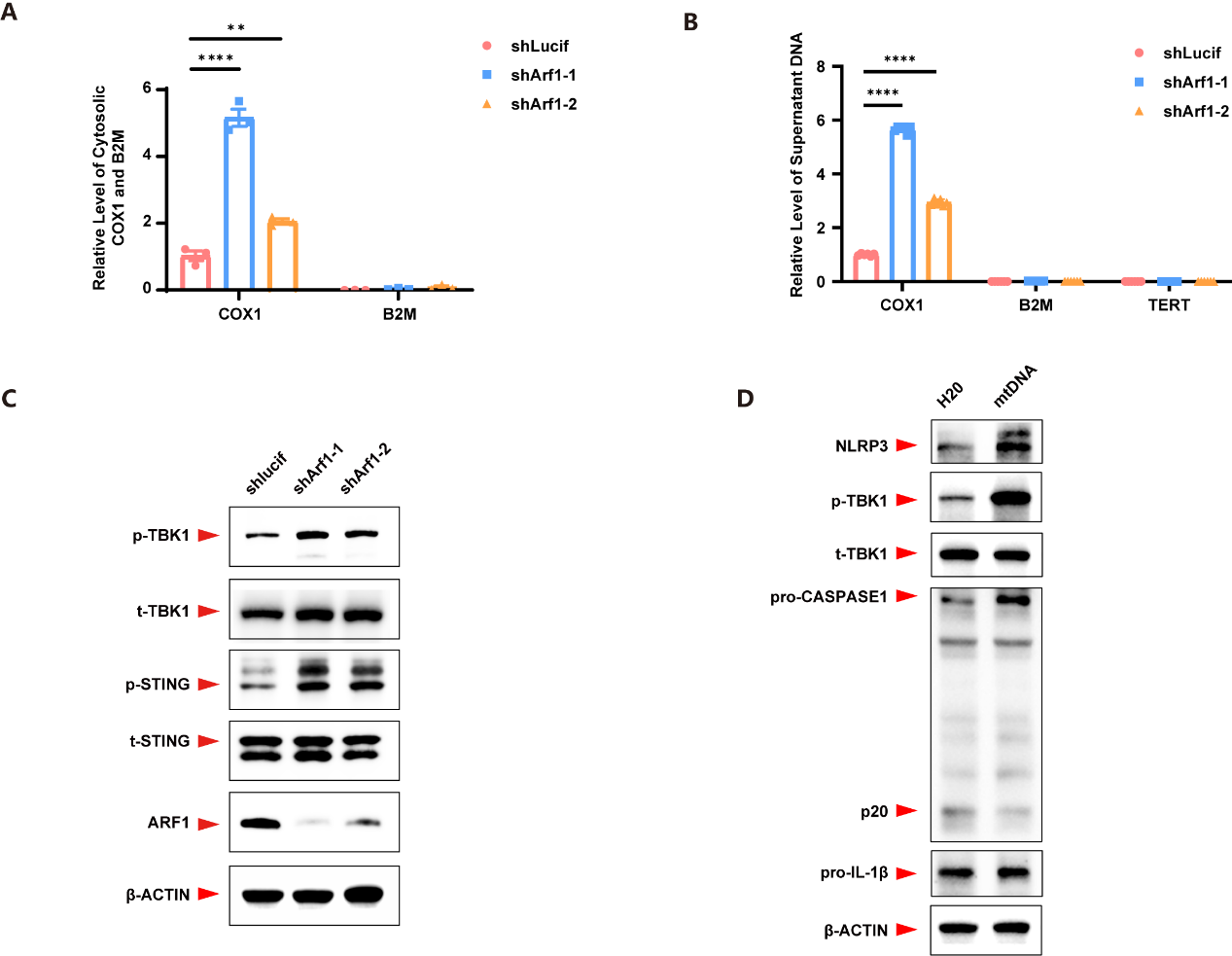
Supplemental Figure 3. Dysfunctional mitochondria induced oxidative damaged mtDNA release and induced microglia inflammation, related to Figure 3.**

1. Relative abundance of cytosolic genomic DNA compared to mtDNA from control or Arf1-deficient N2a cells. n = 3 per group.
2. Relative abundance of supernatant genomic DNA compared to mtDNA from control or Arf1-deficient N2a cells. n = 3 per group.
3. Western blots of control or Arf1-deficient N2a cells.
4. Western blots of Eoc20 cells treated with or without mtDNA transfection as indicated.

Data are shown as the mean ± SEM. *P < 0.05, **P < 0.01. ***P < 0.001. ****P < 0.0001 by two-way ANOVA with Holm Šídák’s multiple comparisons test.

**
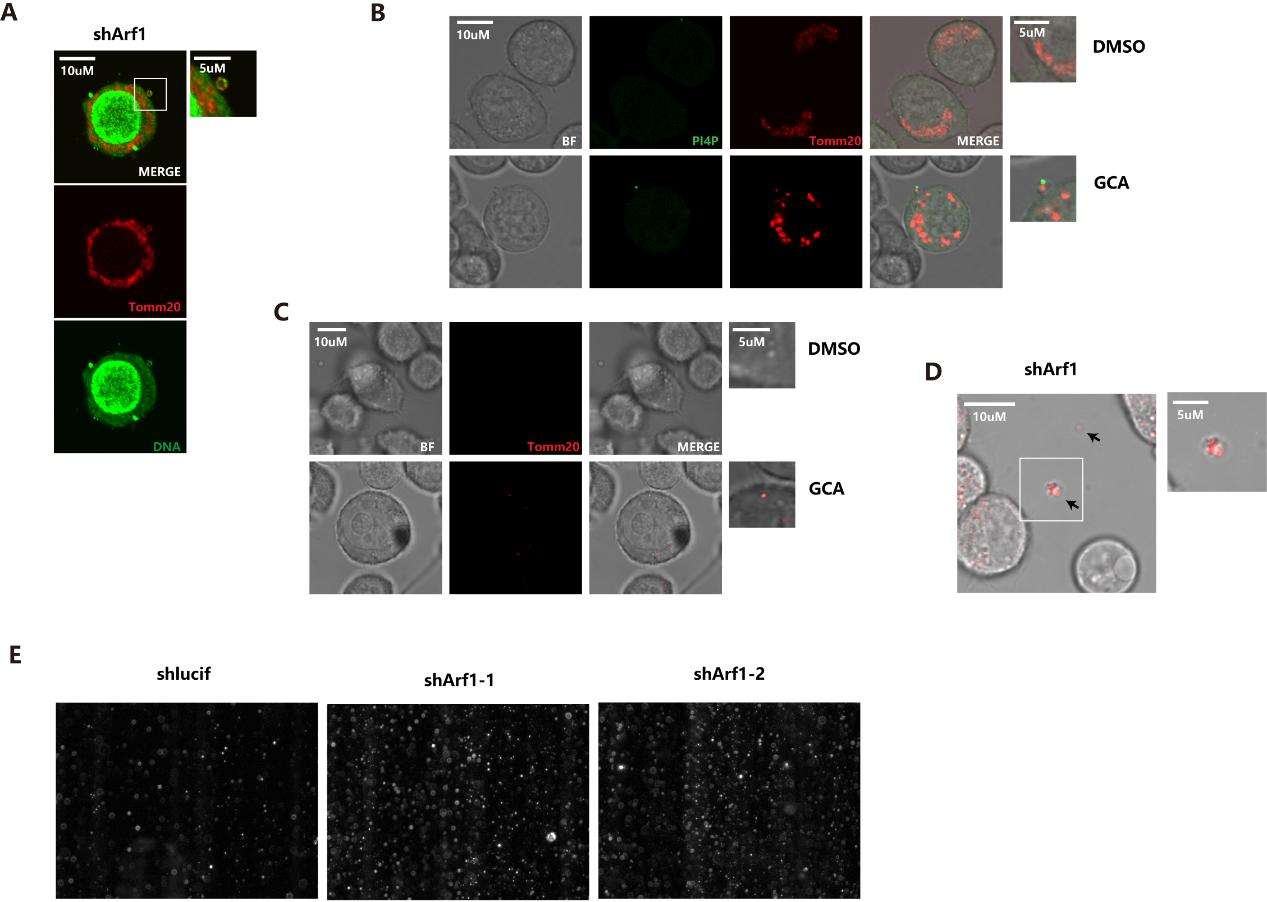
Supplemental Figure 4. Mitochondria-derived exosomes mediated the transportation of mtDNA and induced microglial inflammation, related to Figure 4.**

1. Representative DNA(Green) and Tomm20(Red) image of Arf1-deficient N2a cell.
2. Representative Tomm20-mCherry image of control or GCA-treated N2a cell.
3. Representative Tomm20-mCherry images of Eoc20 cells treated with the conditional medium from control or GCA-treated N2a cell.
4. Representative Tomm20-mCherry contained vesicle images of Arf1-deficient N2a cells.
5. Representative exosomes NTA images of control or Arf1-deficient N2a cells.

**
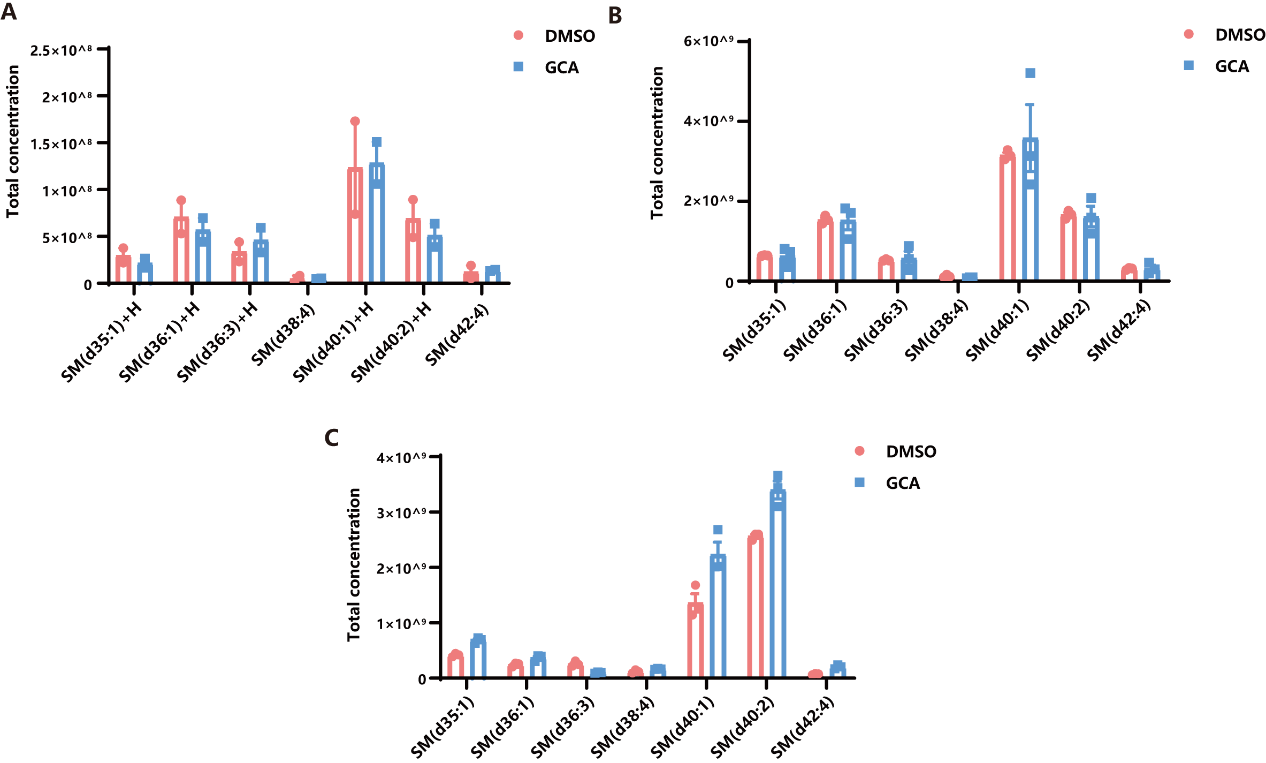
**

**Supplemental Figure 5. Accumulation of sphingolipids in lysosomes causes lysosomal dysfunction and induces MVB formation, facilitating EV secretion, related to Figure 5.**

1. The level of multi-type sphingolipids in ER from control or Arf1-deficient N2a cells. n = 2 per group.
2. The level of multi-type sphingolipids in Nucleus from control or Arf1-deficient N2a cells. n = 3 per group.
3. The level of multi-type sphingolipids in Mitochondria from control or Arf1-deficient N2a cells. n = 2 per group.

Data are shown as the mean ± SEM. *P < 0.05, **P < 0.01. ***P < 0.001. ****P < 0.0001 by two-way ANOVA with Holm Šídák’s multiple comparisons test.

**
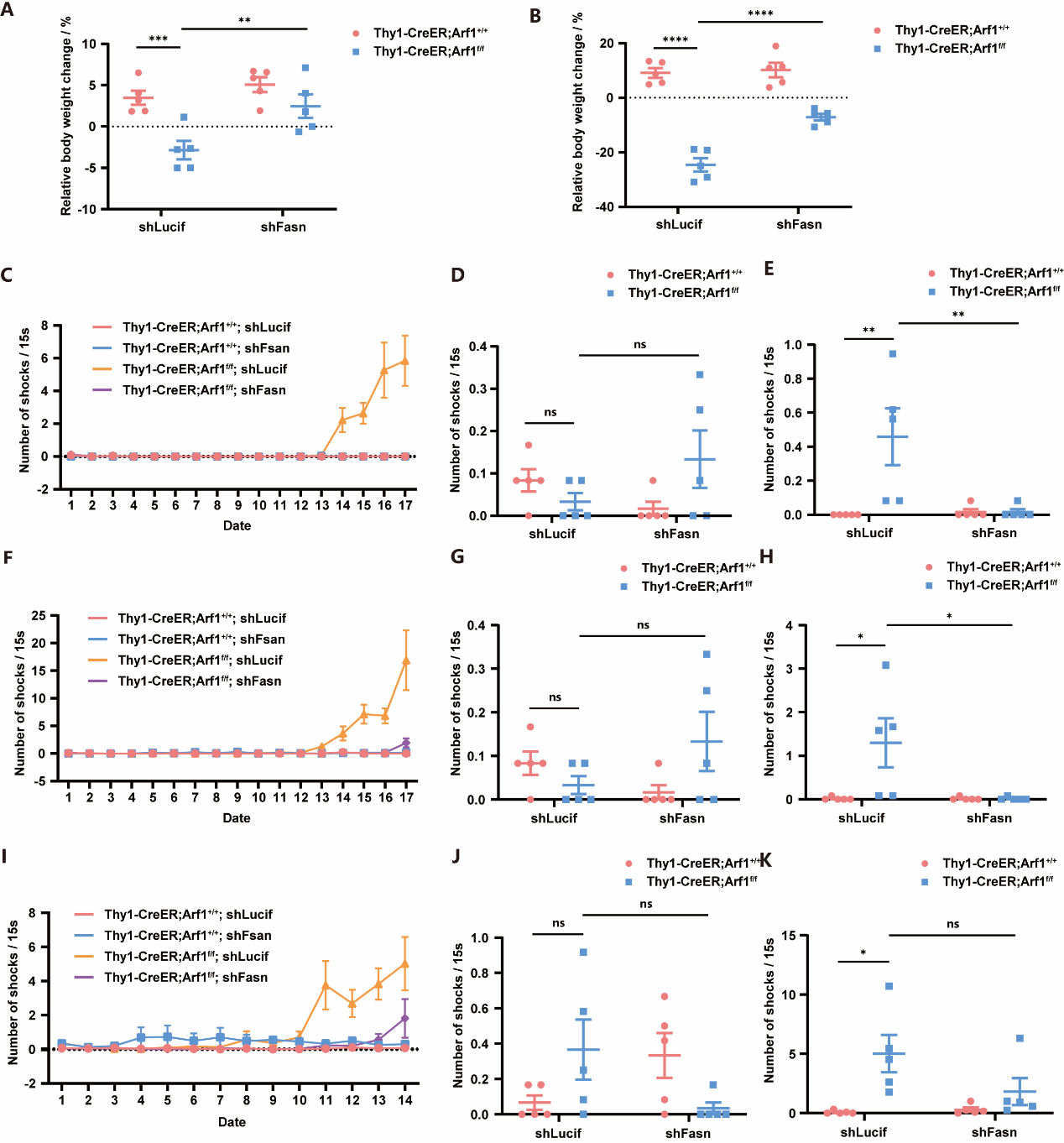
Supplemental Figure 6. Blockade of fatty acid synthesis reverses neuronal degeneration, related to Figure 6.**

1. Relative body weight change of mice compared to the first day on the 9^th^ day. n = 5 per group.
2. Relative body weight change of mice compared to the first day at the 17^th^ day. n = 5 per group.
3. Number of shocks per 15 seconds at the speed of 5m/min. n = 5 per group.
4. Number of shocks per 15 seconds at the speed of 5m/min on the first day. n = 5 per group.
5. Number of shocks per 15 seconds at the speed of 5m/min on the 14^th^ day. n = 5 per group.
6. Number of shocks per 15 seconds at the speed of 10m/min. n = 5 per group.
7. Number of shocks per 15 seconds at the speed of 10m/min on the first day. n = 5 per group.
8. Number of shocks per 15 seconds at the speed of 10m/min on the 13th day. n = 5 per group.
9. Number of shocks per 15 seconds at the speed of 15m/min. n = 5 per group.
10. Number of shocks per 15 seconds at the speed of 15m/min on the first day. n = 5 per group.
11. Number of shocks per 15 seconds at the speed of 15m/min on the 14^th^ day. n = 5 per group.

Data are shown as the mean ± SEM. *P < 0.05, **P < 0.01. ***P < 0.001. ****P < 0.0001 by two-way ANOVA with Holm Šídák’s multiple comparisons test.

**
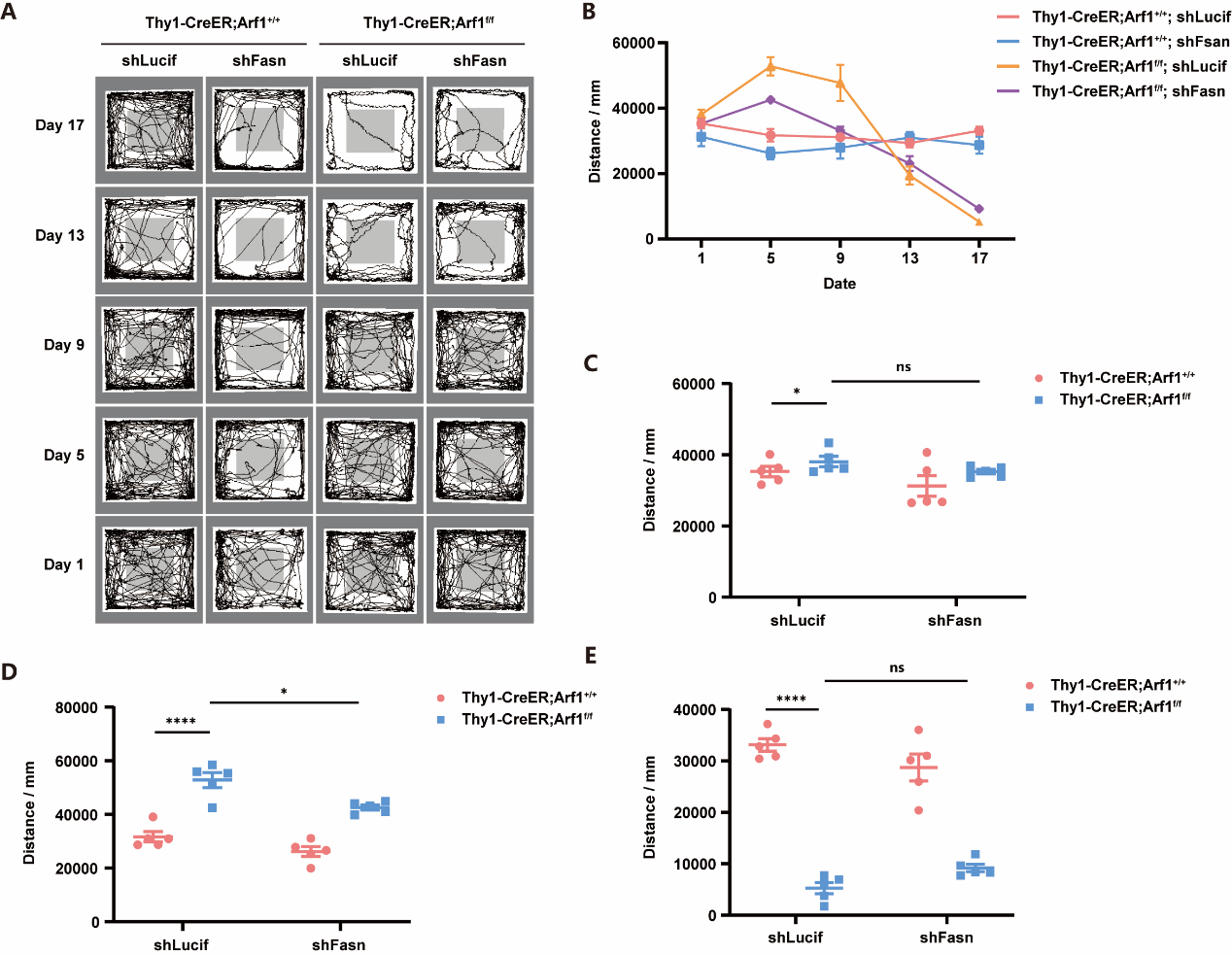
Supplemental Figure 7. Blockade of fatty acid synthesis reverses neuronal degeneration, related to Figure 6.**

1. Movement’s map of mice in an open field.
2. The moved distance of mice in an open field. n = 5 per group.
3. The moved distance of mice in an open field on the first day. n = 5 per group.
4. The moved distance of mice in an open field on the 5^th^ day. n = 5 per group.
5. The moved distance of mice in an open field on the 17^th^ day. n = 5 per group.

Data are shown as the mean ± SEM. *P < 0.05, **P < 0.01. ***P < 0.001. ****P < 0.0001 by two-way ANOVA with Holm Šídák’s multiple comparisons test.

**
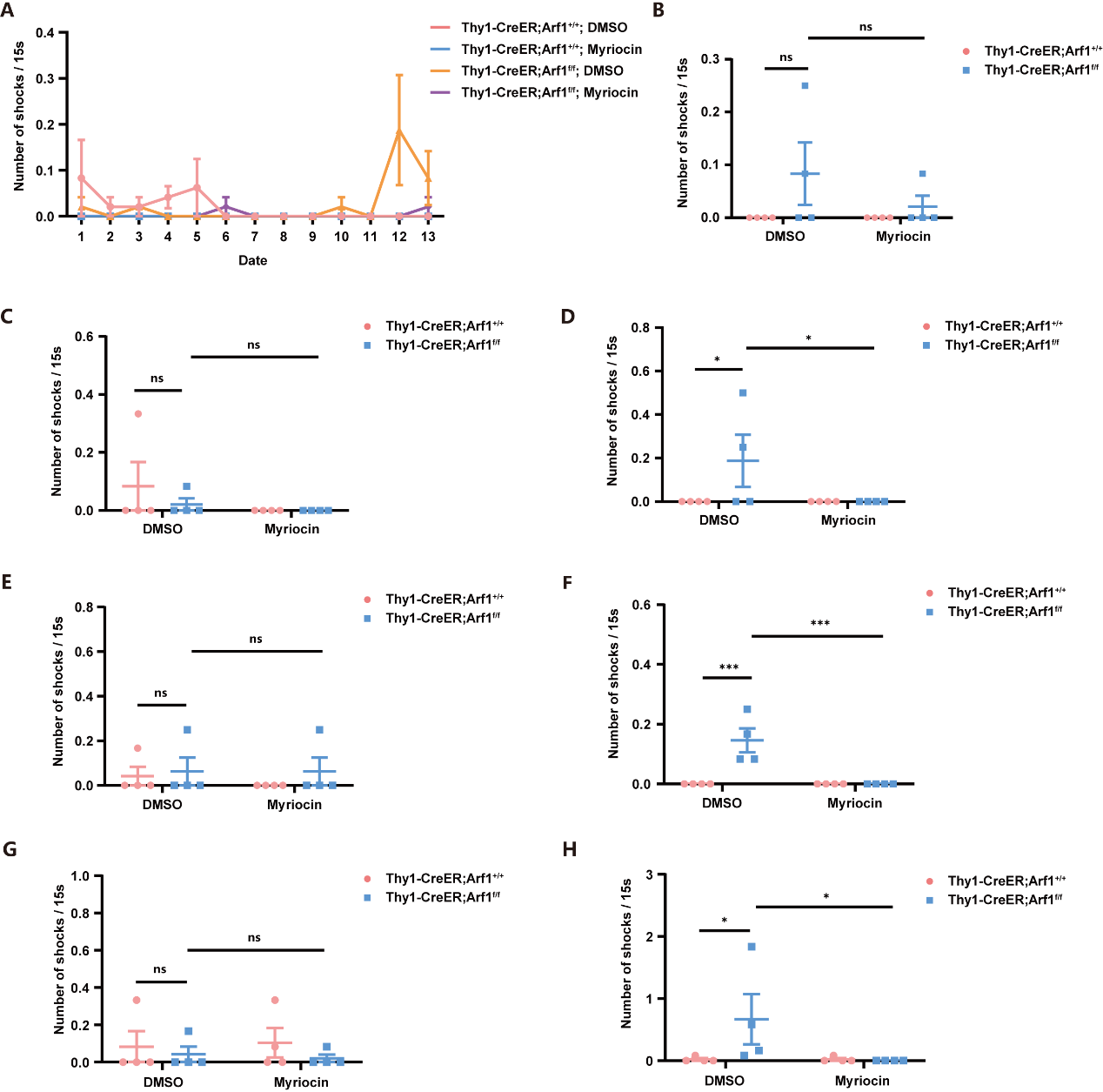
Supplemental Figure 8. Boosting autophagy reverses neuronal degeneration, related to Figure 7.**

1. Number of shocks per 15 seconds at the speed of 5m/min. n = 4 per group.
2. Number of shocks per 15 seconds at the speed of 5m/min on the 13th day. n = 4 per group.
3. Number of shocks per 15 seconds at the speed of 5m/min on the first day. n = 4 per group.
4. Number of shocks per 15 seconds at the speed of 5m/min on the 12th day. n = 4 per group.
5. Number of shocks per 15 seconds at the speed of 10m/min on the first day. n = 4 per group.
6. Number of shocks per 15 seconds at the speed of 10m/min on the 9^th^ day. n = 4 per group.
7. Number of shocks per 15 seconds at the speed of 15m/min on the first day. n = 4 per group.
8. Number of shocks per 15 seconds at the speed of 15m/min on the 7^th^ day. n = 4 per group.

Data are shown as the mean ± SEM. *P < 0.05, **P < 0.01. ***P < 0.001. ****P < 0.0001 by two-way ANOVA with Holm Šídák’s multiple comparisons test.

**
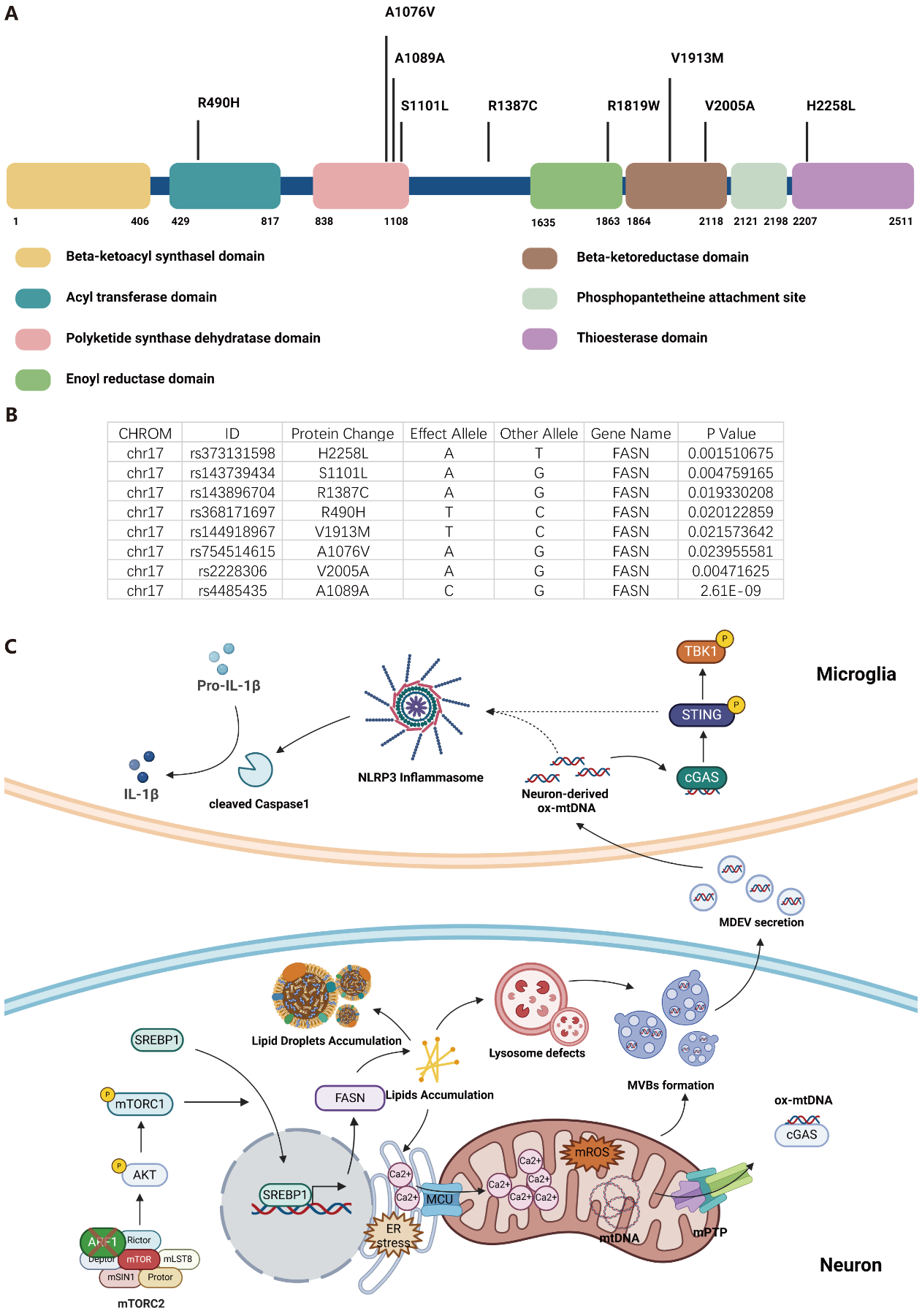
**

**Supplemental Figure 9. FASN variants were discovered in ND patients.**

1. Schematic of the distribution of FASN variants.
2. Detailed information of FASN variants.
3. Schematic of the detailed mechanism of how Arf1 regulated neurodegeneration through modulating lipid metabolism.

**
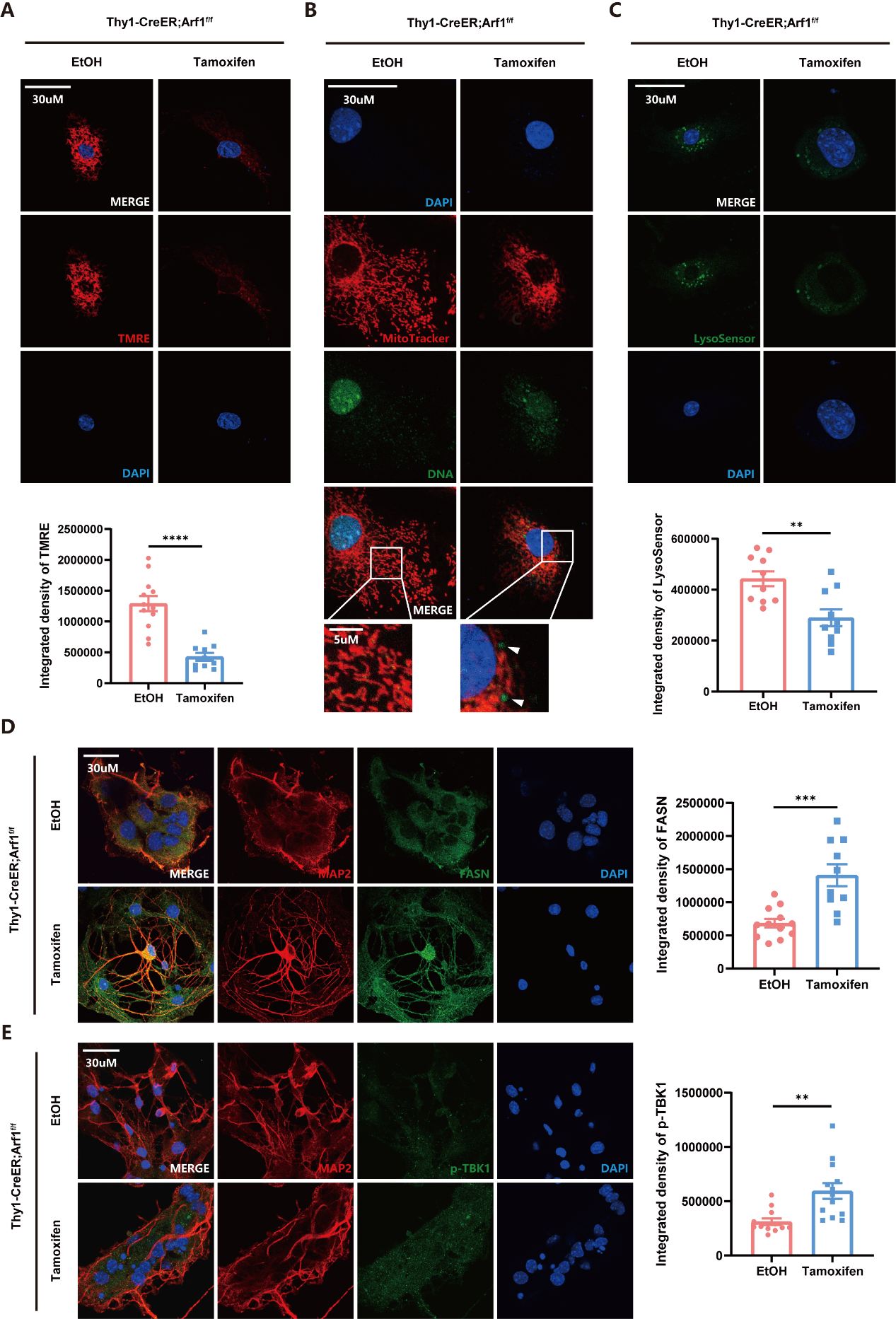
**

**Supplemental Figure 10. Arf1-deficient primary neuron showed impaired neuronal activity.**

1. Representative TMRE images of EtOH or Tamoxifen treated primary neuron obtained from Thy1-CreER / Arf1^f/f^ mice (top); quantification of the integrated fluorescence intensity of TMRE (bottom). n = 12, 10 respectively.
2. Representative DNA (green) and MitoTracker (red) images of EtOH or Tamoxifen treated primary neuron obtained from Thy1-CreER / Arf1^f/f^ mice.
3. Representative LysoSensor images of EtOH or Tamoxifen treated primary neuron obtained from Thy1-CreER / Arf1^f/f^ mice (top); quantification of the integrated fluorescence intensity of LysoSensor (bottom). n = 10 per group.
4. Representative MAP2 (red) and FASN (green) images of EtOH or Tamoxifen treated primary neuron obtained from Thy1-CreER / Arf1^f/f^ mice (top); quantification of the integrated fluorescence intensity of FASN (bottom). n = 12, 10 respectively.
5. Representative MAP2 (red) and p-TBK1 (green) images of EtOH or Tamoxifen treated primary neuron obtained from Thy1-CreER / Arf1^f/f^ mice (top); quantification of the integrated fluorescence intensity of p-TBK1 (bottom). n = 12, 13 respectively.

Data are shown as the mean ± SEM. *P < 0.05, **P < 0.01. ***P < 0.001. ****P < 0.0001 by T test.

**
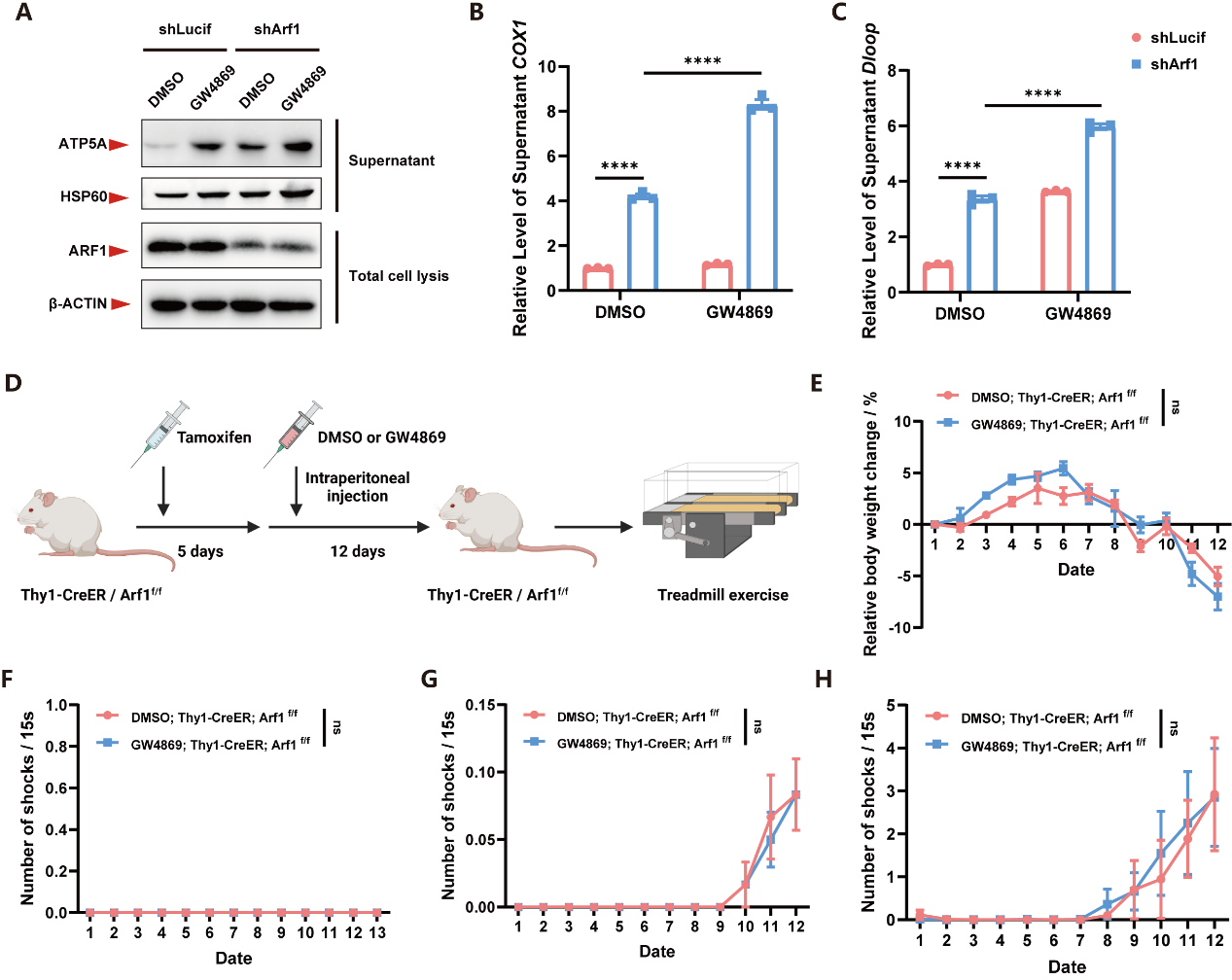
**

**Supplemental Figure 11. Inhibiting exosome secretion is unable to reverses neuronal degeneration.**

1. Western blots of supernatant protein derived from control or Arf1-deficient N2a cells treated with DMSO or GW4869.
2. Relative abundance of supernatant Cox1 from control or Arf1-deficient N2a cells treated with DMSO or GW4869. n = 3 per group.
3. Relative abundance of supernatant Dloop from control or Arf1-deficient N2a cells treated with DMSO or GW4869. n = 3 per group.
4. Schematic of DMSO or GW4869 administration and behavioral experiments of mice.
5. Relative body weight change of mice compared to the first day. n = 5 per group.
6. Number of shocks per 15 seconds at the speed of 5m/min. n = 5 per group.
7. Number of shocks per 15 seconds at the speed of 10m/min. n = 5 per group.
8. Number of shocks per 15 seconds at the speed of 15m/min. n = 5 per group.

Data are shown as the mean ± SEM. *P < 0.05, **P < 0.01. ***P < 0.001. ****P < 0.0001 by two-way ANOVA with Holm Šídák’s multiple comparisons test.

**Table S1. Primers**

| **qPCR primer** | |
| --- | --- |
| **Name** | **Sequence** |
| mt-COX1-F | AACATGAAACCCCCAGCCAT |
| mt-COX1-R | TGGGTGCCCAAAGAATCAGA |
| mt-Dloop-F | TCCTCCGTGAAACCAACAA |
| mt-Dloop-R | AGCGAGAAGAGGGGCATT |
| g-B2M-F | ATGGGAAGCCGAACATACTG |
| g-B2M-R | CAGTCTCAGTGGGGGTGAAT |
| g-TERT-F | CTAGCTCATGTGTCAAGACCCTCTT |
| g-TERT-R | GCCAGCACGTTTCTCTCGTT |
| Acaca-F | ATGGGCGGAATGGTCTCTTTC |
| Acaca-R | TGGGGACCTTGTCTTCATCAT |
| Actin-F | GGCTGTATTCCCCTCCATCG |
| Actin-R | CCAGTTGGTAACAATGCCATGT |
| **Genotyping primer** | |
| Thy1 Forward | TCTGAGTGGCAAAGGACCTTAGG |
| Thy1 Reverse | CGCTGAACTTGTGGCCGTTTACG |
| Arf1-gR | TGCACACACCAAGTACAAGC |
| Arf1-gP3 | TCGGGAGCTGGCACTAAAAA |
| Arf1-gP5 | GGTTTTAAGAGGCCCTGTGTC |
| **shRNA primer** | |
| shArf1-1-F | CCGGTGAGACTGTTGAATACAAGAATCTCGAGATTCTTGTATTCAACAGTCTCTTTTTG |
| shArf1-1-R | aattCAAAAAGAGACTGTTGAATACAAGAATCTCGAGATTCTTGTATTCAACAGTCTCA |
| shArf1-2-F | CCGGTGCAGGGAAGACAACAATTCTACTCGAGTAGAATTGTTGTCTTCCCTGCTTTTTG |
| shArf1-2-R | aattCAAAAAGCAGGGAAGACAACAATTCTACTCGAGTAGAATTGTTGTCTTCCCTGCA |
| shFasn-F | CCGGGCTGGTCGTTTCTCCATTAAACTCGAGTTTAATGGAGAAACGACCAGCTTTTTG |
| shFasn-R | aattCAAAAAGCTGGTCGTTTCTCCATTAAACTCGAGTTTAATGGAGAAACGACCAGC |
